# iGATE analysis improves the interpretability of single-cell immune landscape of influenza infection

**DOI:** 10.1172/jci.insight.172140

**Published:** 2024-05-30

**Authors:** Brett D. Hill, Andrew J. Zak, Sanjeev Raja, Luke F. Bugada, Syed M. Rizvi, Saiful B. Roslan, Hong Nhi Nguyen, Judy Chen, Hui Jiang, Akira Ono, Daniel R. Goldstein, Fei Wen

**Affiliations:** 1 Department of Chemical Engineering,; 2 Program in Immunology,; 3 Department of Internal Medicine,; 4 Department of Biostatistics, and; 5 Department of Microbiology and Immunology, University of Michigan, Ann Arbor, Michigan, USA.

**Keywords:** Immunology, Virology, Bioinformatics, Influenza, Proteomics

## Abstract

Influenza poses a persistent health burden worldwide. To design equitable vaccines effective across all demographics, it is essential to better understand how host factors such as genetic background and aging affect the single-cell immune landscape of influenza infection. Cytometry by time-of-flight (CyTOF) represents a promising technique in this pursuit, but interpreting its large, high-dimensional data remains difficult. We have developed a new analytical approach, in silico gating annotating training elucidating (iGATE), based on probabilistic support vector machine classification. By rapidly and accurately “gating” tens of millions of cells in silico into user-defined types, iGATE enabled us to track 25 canonical immune cell types in mouse lung over the course of influenza infection. Applying iGATE to study effects of host genetic background, we show that the lower survival of C57BL/6 mice compared with BALB/c was associated with a more rapid accumulation of inflammatory cell types and decreased IL-10 expression. Furthermore, we demonstrate that the most prominent effect of aging is a defective T cell response, reducing survival of aged mice. Finally, iGATE reveals that the 25 canonical immune cell types exhibited differential influenza infection susceptibility and replication permissiveness in vivo, but neither property varied with host genotype or aging. The software is available at https://github.com/UmichWenLab/iGATE.

## Abstract 2

iGATE comprehensively profiles user-defined immune cell types with regards to their abundance, activation, cytokine profile, susceptibility to influenza infection, and permissiveness to influenza replication in vivo.

## Graphical abstract

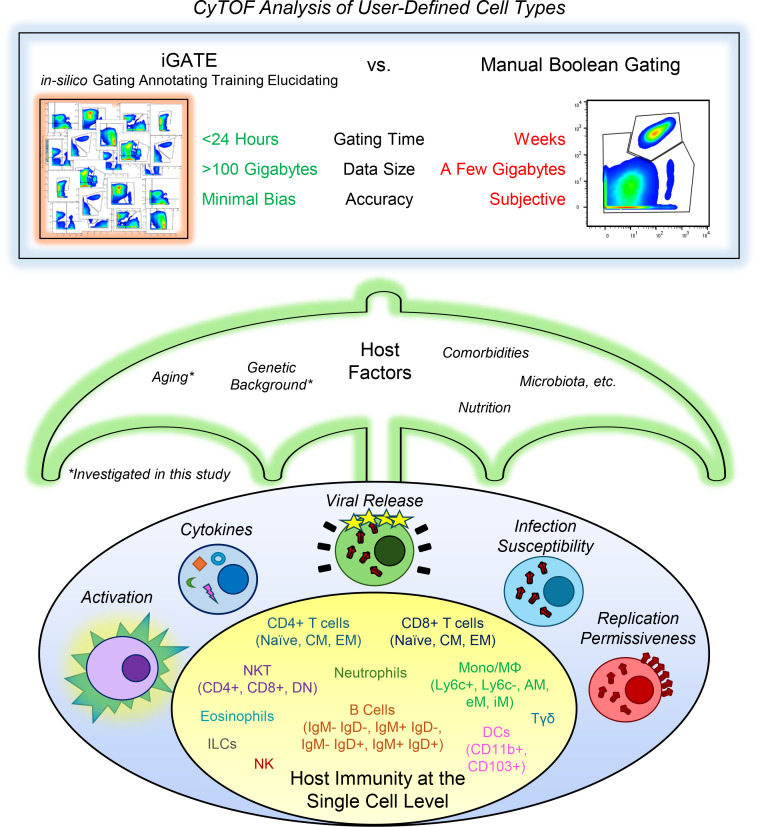

## Introduction

Despite the development of a substantial infrastructure for influenza strain surveillance and annual vaccine manufacture, seasonal influenza still causes millions of cases of severe illness and 290,000–650,000 deaths per year worldwide (1). Although vaccination is the primary means of preventing influenza infection, overall influenza vaccine effectiveness is only moderate and varies highly from year to year, ranging from 10% to 60% (2). The National Institute of Allergy and Infectious Diseases (NIAID) recently established a plan for universal influenza vaccine development, aiming to provide effective and durable protection against diverse influenza strains without the annual vaccination requirement (3).

To this end, most universal vaccination approaches aim to elicit broadly reactive antibodies (4, 5) and T cells (6) that recognize cross-reactive influenza epitopes. However, understanding why some individuals develop protective immunity after infection or vaccination while others do not is just as crucial. For example, only half of young adults infected with influenza were protected from reinfection by the same viral strain approximately 1 year later (7). Many host factors such as aging, comorbidities, nutrition, microbiota, and genetics greatly influence the immune system and impact influenza disease severity and vaccine efficacy (8–10). Aging, in particular, leads to increased susceptibility to infections due to immunosenescence (11), and those above 65 often have difficulty mounting protective immune responses after vaccination and consequently account for 70%–90% of all influenza-related deaths (12). Beyond aging, vaccine effectiveness is still surprisingly modest in low-risk age groups for well-matched vaccines (13), suggesting that other host factors affect the immune response. In particular, genetic variation has been shown to play an important role in the susceptibility of both humans and mice to a variety of diseases, including influenza (14–16). Thus, a deeper understanding of how host factors shape immune responses is critical for developing equitable vaccines and treatments that are effective across all demographics.

However, due to the incredible complexity of the immune landscape, it has been challenging to comprehensively characterize the immune response following infection with influenza or other pathogens, let alone the effect of host factors. Technological limitations have restricted studies to either bulk measurements (e.g., serum antibody levels) or highly focused investigations into specific immune components (e.g., individual cellular subsets). The recent advent of omics approaches now allows high-dimensional measurements to be performed on an unprecedented number of biological variables (9). These powerful systems-immunology approaches, combined with computational and machine-learning tools to extract meaning from the resulting high-dimensional data sets, have revealed new insights into methods of stimulating and shaping the immune response (17, 18), ultimately enabling a more predictive approach to vaccine design.

In this work, we apply cytometry by time-of-flight (CyTOF) to comprehensively investigate how host genetic background (C57BL/6 vs. BALB/c) and aging (2 vs. 18 months) affect the immune response to influenza infection in the mouse lung. CyTOF is a promising single-cell immune profiling technique that allows for simultaneous detection of approximately 40 markers on single cells (19). However, due to data set complexity and computational limitations, the field has largely avoided conventional Boolean gating, turning instead toward unsupervised approaches (20). While excellent for exploratory, big-picture analyses, it is often challenging to connect the results of these unsupervised approaches back to canonical cell types, which is necessary to fully interpret immunological mechanisms (20–22). To improve the interpretability of large, high-dimensional CyTOF data sets, we developed in silico gating annotating training elucidating (iGATE), a new analytical approach that enabled rapid, automated classification of 29 million cells into 25 canonical immune cell types with high accuracy (98.1% on average). By tracking not only the activation markers and cytokines, but also intracellular and surface expression of the influenza viral protein hemagglutinin (HA) across all cell types, this study presents the most comprehensive cytometry-based single-cell analysis of the influenza immune landscape in mice to date. Analysis of the resulting data reveals marked discrepancies in immune cell infiltration, activation, cytokine environment, and viral activity due to host genetic background and aging. Taken together, our results show that CyTOF, combined with iGATE, permits a systems-level exploration of canonical immune cell types, providing cellular and molecular insights into the role of host factors during influenza infection.

## Results

### iGATE enables automated and accurate classification of large high-dimensional CyTOF data into user-defined canonical immune cell types.

**Figure 1:**
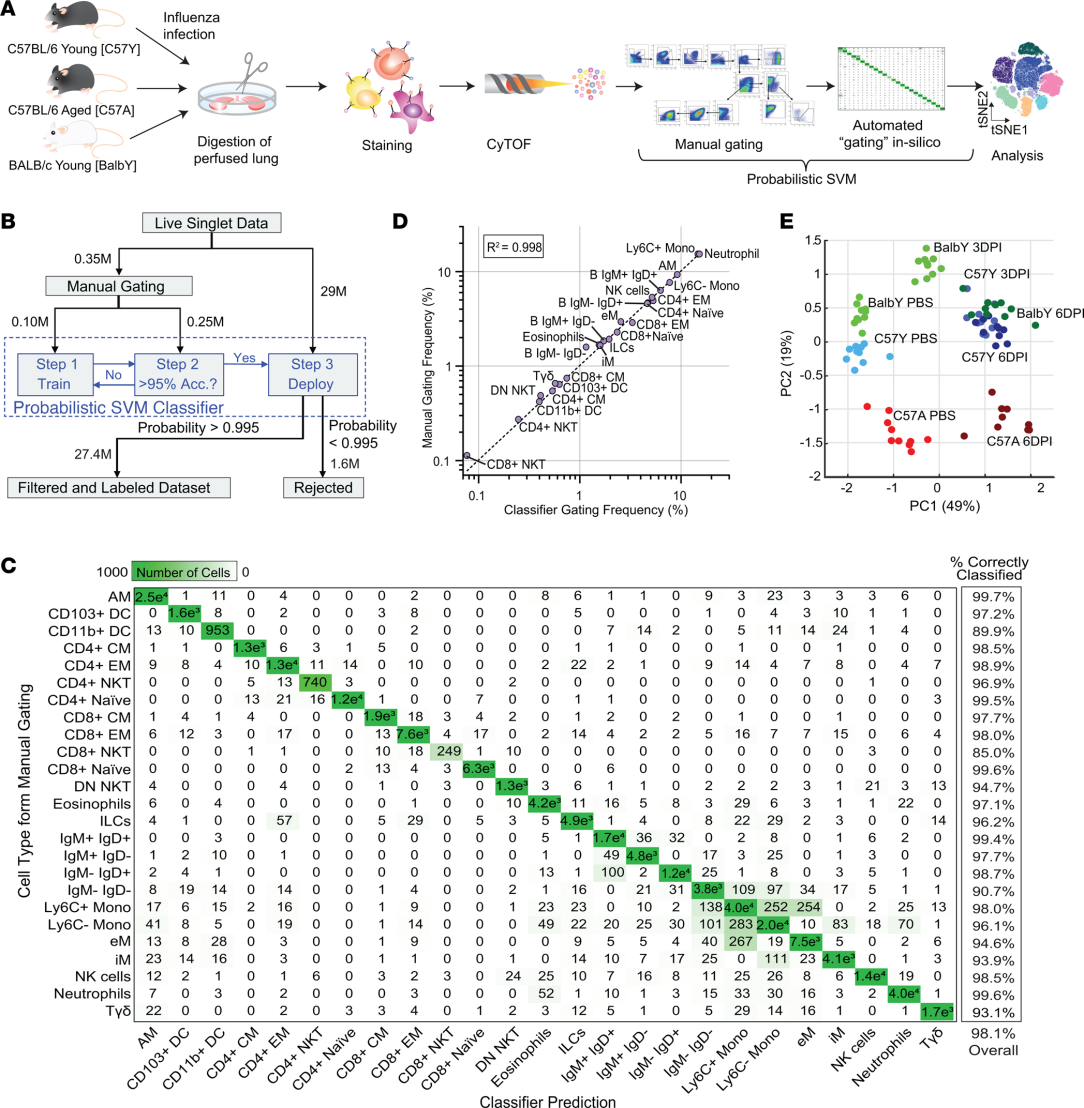
Automated classification of 29-million-cell data set using iGATE. (**A**) Young C57BL/6 (C57Y), aged C57BL/6 (C57A), and young BALB/c (BalbY) mice were intranasally infected with H1N1 A/PR/8/34 virus or treated with PBS. Perfused lungs were harvested 3 or 6 days post infection (DPI) (*n* = 10 per group), stained with a 40-marker panel, and analyzed by CyTOF. After pregating to obtain live singlets, CD45^+^ cells were classified into 25 immune cell types using a probabilistic SVM classifier. See Table 1 for cell type definitions and Supplemental Material for a complete list of abbreviations. (**B**) Analysis pipeline for the iGATE probabilistic SVM classifier. (**C**) Confusion matrix comparing probabilistic SVM classification to manual gating using a 250,000 cell validation data set sampled from all groups. The accuracies shown on the right were calculated as the proportion of correctly classified cells out of the total cells for each cell type (per-class accuracy) or for all cells (overall accuracy). (**D**) Comparison of manual gating and SVM classifier gating reveals high accuracy of SVM classifier. (**E**) Principal component analysis (PCA) of the 25 cell type frequencies differentiates samples based on infection status, age, and genotype.

To better understand how host genetic background and aging affect the influenza immune response, we compared influenza infection in young (2 month) C57BL/6 mice (C57Y) to both young (2 month) BALB/c (BalbY) and aged (18 month) C57BL/6 (C57A) mice (Figure 1A). Viral challenge experiments showed that infection with 2.0 × 10^4^ PFU of influenza H1N1 A/PR/8/34 (PR8) virus resulted in 0% survival for C57A, 14% for C57Y, and 29% for BalbY (Supplemental Figure 1; supplemental material available online with this article; https://doi.org/10.1172/jci.insight.172140DS1). Using different influenza virus strains and a range of doses, many prior studies also showed increased mortality of C57A (23–26) and better survival of BalbY (27–31) compared with C57Y. To investigate the mechanisms of this differential protection, lungs from PBS-treated mice (*n* = 10) and PR8-infected mice (1.2 × 10^4^ PFU) at 3 days post infection (3DPI) or 6DPI (*n* = 10 for each day) were perfused, harvested, processed (see Methods), and stained with a 40-marker panel that was designed and validated to identify 25 canonical immune cell types as listed in Table 1 from both myeloid and lymphoid lineages along with their expression of various intracellular and surface markers for activation, cytokines, and influenza-specific proteins (Supplemental Table 1). After gating to remove debris, dead cells, and doublets (Supplemental Figure 2A), a total of 29 million CD45^+^ live singlet cells remained across all mouse samples for single-cell analysis.

Since unsupervised data analysis approaches (e.g., Phenograph, SPADE, and CITRUS) are not based on predetermined, well-defined phenotypes, they can instead identify intermediate populations that are difficult to interpret or miss small but relevant subpopulations (20–22). Therefore, supervised analyses can be advantageous for better extracting biological meaning in well-defined systems by tracking changes in canonical cell populations. Given that our panel was designed to identify cellular subsets known to play crucial roles in the immune response to influenza infection, we pursued a supervised approach to data analysis. The most traditional form of supervised analysis, manual Boolean gating of cells on biaxial plots into user-defined subsets, is highly time consuming, subjective, and not scalable to very large data sets such as those generated by CyTOF. To address these shortcomings, we developed iGATE to achieve automated cell labeling based on a support vector machine (SVM) classifier (32). SVM learns hyperplanes (i.e., linear decision boundaries) that optimally divide the data by user-defined classes. Following training and validation, the classifier can be deployed on large data sets in a computationally efficient manner, enabling rapid classification into cellular subsets. The complex nature of the single-cell immune landscape inevitably leads to the presence of numerous noncanonical and boundary cell populations, a situation that is exacerbated in mass cytometry due to the large number of detected markers. To accommodate this complexity, we modified the conventional SVM classifier by incorporating a rejection option (33) following classification based on posterior probabilities (see Supplemental Methods). In this scheme, the posterior probability of each cell belonging to its predicted class is computed, and cells not meeting a predefined threshold are rejected and placed into an “other” class. Such an approach allows enhanced classification control by fine-tuning the probability threshold to balance accuracy and the rate of rejected cells (Supplemental Figure 3).

To establish a data set for classifier training and validation, 350,000 cells were first randomly sampled from the entire data set of 29 million cells such that an equal number of cells were taken from each treatment group. This small data set was then manually gated to define the 25 immune cell types (Supplemental Figure 2B), and randomly sampled to generate the training (100,000 cells) and validation (the remaining 250,000 cells) data sets (Figure 1B). The trained classifier achieved an average validation accuracy of 98.1%, and the confusion matrix reveals that most per-class accuracies are above 95%, with most large off-diagonal terms corresponding to misclassifications between similar cellular subsets such as Ly6C^+^ monocytes (Ly6C^+^ Mos), Ly6C^–^ Mos, and exudative macrophages (eMs) (Figure 1C). Note that no cells were rejected based on posterior probabilities in these validation results. Comparison of cell-type frequencies from manual gating and probabilistic SVM classification revealed that the classifier achieves a high degree of accuracy across a frequency range encompassing more than 2 orders of magnitude (Figure 1D), validating its use for identifying both abundant and rare cell populations. Deploying the trained classifier on the entire 29 million cells with a posterior probability threshold of 0.995 yielded a cell rejection rate of approximately 6% (Supplemental Figure 3), which is consistent with the 5%–10% rejection rate from manual gating. Rejected cells were found to exist predominantly on gate boundaries (Supplemental Figure 4), suggesting that the probabilistic approach can effectively identify transitional or noncanonical cell populations. When the same data set was analyzed using the unsupervised method Phenograph (performed in Cytobank, https://cytobank.org), it failed to identify 10 out of the 25 canonical cell types identified by iGATE and instead identified many intermediate populations that were difficult to interpret (Supplemental Figure 5, A–C). Moreover, comparing cell types that were identified by both Phenograph and iGATE, the latter yielded more homogeneous populations, with far fewer incorrectly classified cells (Supplemental Figure 5, D and E). Therefore, the capability of iGATE to rapidly and accurately classify large data sets into user-defined cell types can improve the interpretability of complex single-cell immune landscapes.

To gain a preliminary, systems-level understanding of the data, the frequencies of the 25 cell types identified by the probabilistic SVM classifier for all samples were subjected to principal component analysis (PCA). As shown in Figure 1E, principal component 1 (PC1), which explains nearly 50% of the data variation, correlates with infection status, while PC2, accounting for an additional 19% of data variation, broadly correlates with age. Furthermore, variance is seen in the clustering of young mouse samples due to genotype (Supplemental Figure 6). These results demonstrate that the combination of the chosen antibody panel, manual gating scheme, and probabilistic SVM classifier effectively identifies cell populations relevant to genotype, aging, and infection status in the influenza immune landscape, while the tight intragroup clustering of samples attests to the reproducibility of our experimental methods.

### Deep profiling of immune subsets during influenza infection in C57Y mouse lung.

**Figure 2:**
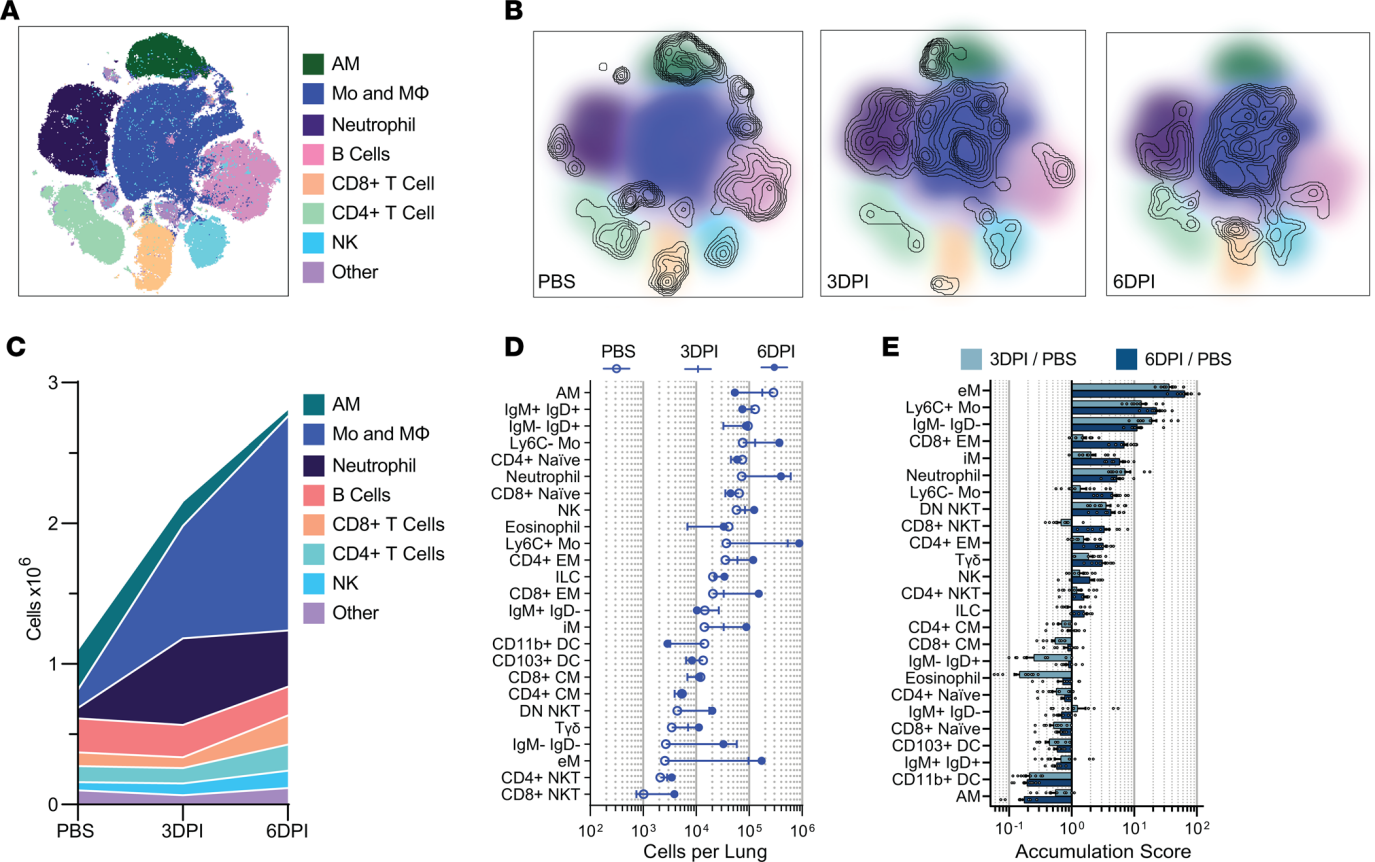
Dynamic changes in immune cell makeup during influenza virus infection in C57Y mouse lung. (**A**) Immune landscape of influenza-infected lung in C57Y mice revealed by viSNE analysis. (**B**) Density plots representing cellular distribution of PBS-treated, 3DPI, and 6DPI C57Y mice overlaid on viSNE plot reveal substantial changes in immune cell composition over the course of infection. (**C**) Absolute cell counts of broad cell classifications indicate substantial immune infiltration. (**D**) Mean absolute cell counts of 25 cellular subsets at indicated conditions. Cell types are ordered based on PBS samples. (**E**) Mean fold change in absolute cell counts for each cell type at 3DPI or 6DPI compared to PBS (accumulation score). Cell types are ordered based on accumulation score at 6DPI. All data represent *n* = 10 per group. All error bars represent ±SEM.

We first comprehensively characterized the immune response in the lung of C57Y mice as a baseline for subsequent comparison to that of BalbY and C57A mice. A systems-level map of the immune landscape was generated via viSNE (34) (Figure 2A) based on preselected phenotypic markers (Supplemental Figure 7). When the viSNE map is colored by the probabilistic SVM cellular classifications, the cell types separate into distinct areas, demonstrating that these 2 approaches are in concordance with each other. To gain a preliminary understanding of cellular composition changes over the course of influenza infection, the viSNE map was overlaid with cell density contour plots representing PBS-treated, 3DPI, or 6DPI C57Y samples (Figure 2B). While tissue-resident alveolar macrophage (AM) frequencies (dark green) were substantially diminished over the course of influenza infection, neutrophils (purple) and non–tissue-resident monocytes/macrophages (dark blue) were substantially increased, indicating rapid lung inflammation.

Next, cell-type frequencies were combined with lung cell counts to obtain absolute changes in lung cellular makeup over the indicated time points (Figure 2C). Significant immune cell infiltration was observed, with CD45^+^ cells doubling by 3DPI (*P* < 0.05) and nearly tripling by 6DPI (*P* < 0.0001) compared with PBS-treated mice. Inflammatory monocytes, macrophages, and neutrophils primarily drove the increase in cellularity, whereas lymphoid lineage cells contributed relatively little to the total cellular accumulation. Furthermore, we observed that the kinetics of cellular accumulation differ by cell type. For example, the neutrophil count peaked at 3DPI, while monocytes and macrophages continued to accumulate through 6DPI.

Following this high-level analysis, the immune landscape was divided into the 25 canonical cellular subsets (definitions in Table 1) to gain a high-resolution snapshot of the changing immune landscape during influenza infection. The absolute cell subset counts per mouse lung spanned nearly 4 orders of magnitude, and the increased resolution revealed several additional cellular subsets undergoing dynamic changes in response to influenza infection such as CD4^+^ and CD8^+^ effector memory (EM) T cells, eosinophils, and dendritic cell (DC) subsets (Figure 2D). To better observe changes in cellular accumulation or depletion, the fold changes in absolute cell counts compared to PBS-treated mice (hereafter referred to as the accumulation score) were computed for all subsets (Figure 2E). At 3DPI, inflammatory phagocytic cell types showed high accumulation scores (Figure 2E, light blue), with eMs increasing 38-fold, Ly6C^+^ Mos increasing 15-fold, and neutrophils increasing 9-fold. More modest increases were observed in other innate immune cell subsets, including interstitial macrophages (iMs), Ly6C^–^ Mos, and natural killer (NK) cells. In contrast, many cellular subsets involved in the adaptive immune response were diminished in the lung, such as T cell subsets (except EM populations), DCs, and IgD^+^ B cells.

At 6DPI, accumulation of inflammatory cell types waned, whereas strong increases in Ly6C^–^ Mos (5-fold), iMs (6-fold), and CD8^+^ EM T cells (7-fold) were observed (Figure 2E, dark blue). Ly6C^–^ Mos and iMs have been suggested to play a role in tissue repair, as opposed to their inflammatory Ly6C^+^ Mo and eM counterparts (35), and therefore indicate a transition to an inflammation resolution response. The accumulation of CD8^+^ EM T cells indicates an influenza-specific adaptive immune response. While the aforementioned changes in cellular subsets are largely consistent with the literature (36), we unexpectedly observed large changes in Tγδ cells and eosinophils (Figure 2E), cell types not traditionally associated with clearance of viral infections. In particular, eosinophils displayed complex behavior, decreasing nearly 10-fold at 3DPI before rebounding back to basal levels by 6DPI (Figure 2E).

**Figure 3:**
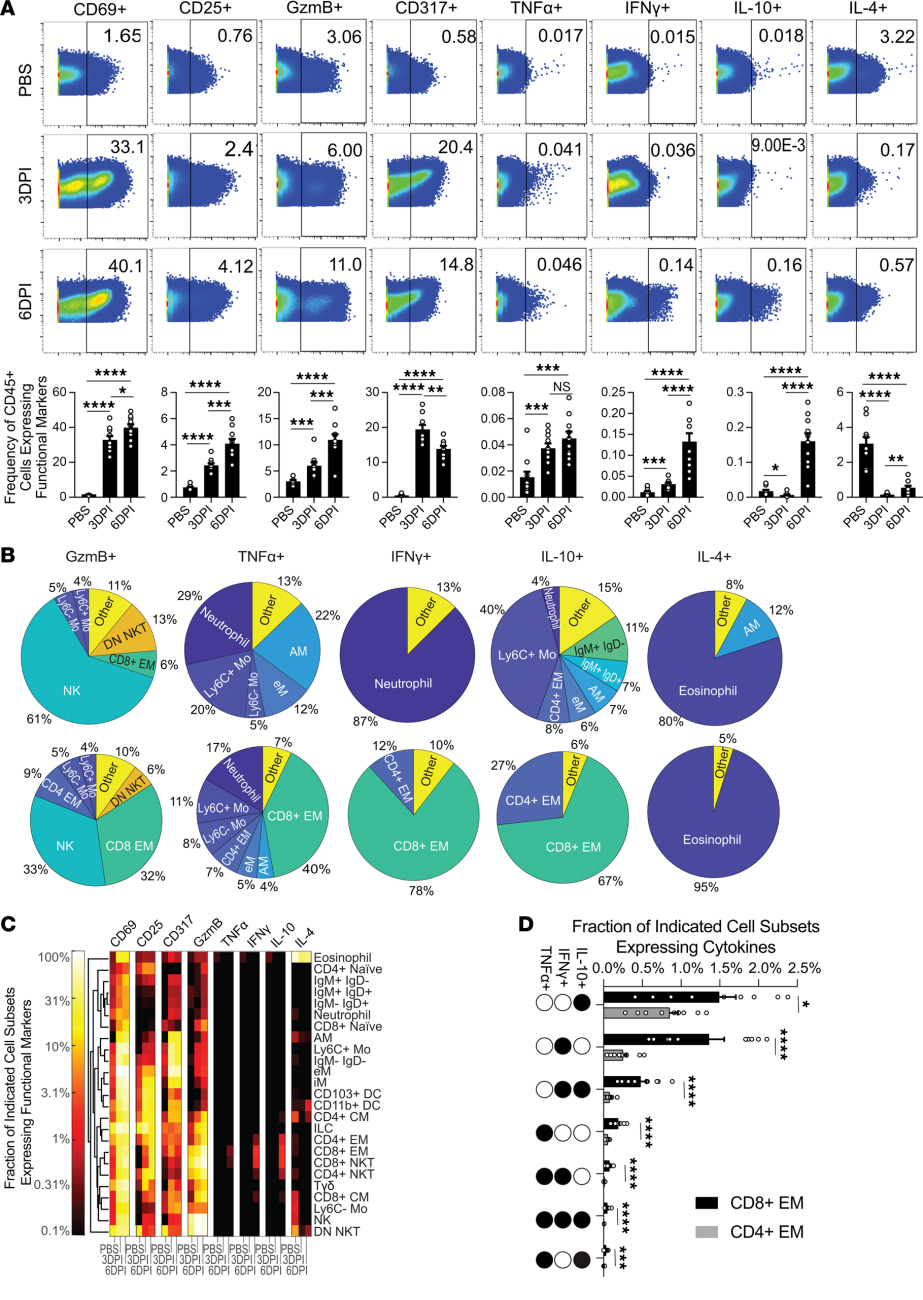
Dynamic phenotypic and functional changes in 25 canonical immune cell types during influenza virus infection in C57Y mouse lung. (**A**) Biaxial plots of CD45 expression versus functional marker expression for CD45^+^ live singlets. Bar charts summarize mean marker frequency out of total CD45^+^ cells. (**B**) Contribution of each cell type to total cytokine positive cells. Contributions of less than 4% are grouped into “other.” (**C**) Mean fraction of functional marker expression on each cell type. (**D**) Coproduction of cytokines by CD4^+^ and CD8^+^ EM T cells at 6DPI. All data represent *n* = 10 per group. All error bars represent ±SEM. Statistical comparisons were computed by 1-way ANOVA followed by post hoc Tukey’s pairwise comparisons (**A**) or 2-sided Student’s *t* test with FDR = 10% (**D**). *q < 0.10; **q < 0.01; ***q < 0.001; ****q < 0.0001.

In addition to the granular changes in cellular composition, cellular populations undergo dynamic alterations in marker expression to interact and respond to their environment. Thus, we next evaluated changes in activation (CD69 and CD25), cytokine (granzyme B [GzmB], TNF-α, IFN-γ, IL-10, IL-4), and IFN-inducible (CD317, i.e., tetherin) markers in bulk CD45^+^ cells (Figure 3A). Positive gates were set according to functional marker expression in PBS-treated samples (Figure 3A, top row). As expected, the frequency of CD69^+^, CD25^+^, GzmB^+^, and CD317^+^ cells significantly increased at 3DPI and 6DPI compared with PBS-treated mice, demonstrating cellular activation and the initiation of effector functions in response to viral infection. Notably, we did not stimulate these cells ex vivo (see Methods); thus, these results provide a real-time snapshot of in vivo cytokine production in single cells, which has rarely been investigated. PBS-treated C57Y mice predominantly expressed IL-4, indicating the basal level lung environment is skewed toward a Th2-type bias in C57Y mice. Upon infection, IL-4^+^ cells decreased precipitously, giving rise to increased levels of the Th1 inflammatory cytokines TNF-α and IFN-γ. IL-10, an antiinflammatory cytokine, was largely absent until 6DPI. Given that IL-10 is known to play a critical role in protecting the host from tissue damage (37), its upregulation at 6DPI further indicates the induction of the inflammation resolution response. Notably, the frequency of cytokine-positive cells measured by CyTOF largely mirrors cytokine levels in bronchoalveolar lavage (BAL) measured by ELISA (Supplemental Figure 8 and ref. 24), confirming that TNF-α, IFN-γ, and IL-10 remain near basal levels until 6DPI. Thus, cytokine detection via CyTOF intracellular staining accurately reveals the cytokine milieu of the infected lung, with the added ability detailed below to identify the cellular sources of cytokine production.

As shown in Figure 3B, monocytes, macrophages, and neutrophils made up the majority of TNF-α^+^, IFN-γ^+^, and IL-10^+^ cells at 3DPI. In contrast, CD8^+^ EM and CD4^+^ EM T cells took over as the major producers of TNF-α (47%), IFN-γ (90%), and IL-10 (94%) at 6DPI, demonstrating a transition in cytokine production from the innate to the adaptive arm of the immune response. To identify changes in functional marker expression in rarer cell types, we next assessed the fraction of each of the 25 canonical immune cell types expressing each functional marker (Figure 3C). Activation markers CD69 and CD25 and type I IFN–inducible marker CD317 were promiscuously expressed, undergoing upregulation in virtually all cell types following infection. Increases in GzmB expression were also detected in nearly all immune subsets by 6DPI, with the highest fractions (>10%) occurring in NKT, NK, and T cell subsets. Note that GzmB expression, while traditionally believed to be exclusive to NK and cytotoxic T cells, has recently been discovered in many immune and nonimmune subsets, with newly identified roles in cellular signaling, chemotaxis, and cytokine release, among others (38). More studies are needed to corroborate the findings that nearly all immune subsets express GzmB and further investigate the roles of GzmB during viral infection. In contrast with GzmB, only a few cell types, including CD4^+^ and CD8^+^ EM T cells and CD4^+^ and CD8^+^ NKT cells, had fractions greater than 0.1% expressing TNF-α, IFN-γ, or IL-10, suggesting a much more restricted expression profile. Although representing a relatively small frequency of all cells (Figure 3A), a large fraction (>80%) of eosinophils (Figure 3, B and C) produced IL-4 throughout the course of infection despite the clear Th1-type cytokine environment. While the significance of this is unknown, the observed IL-4 production may help regulate the strong Th1-type response in conjunction with IL-10 (39).

Given the considerable TNF-α, IFN-γ, and IL-10 cytokine production by both CD4^+^ and CD8^+^ EM T cells at 6DPI (Figure 3, B and C), we further investigated the combinatorial cytokine production in these 2 cellular subsets. While CD8^+^ EM T cells demonstrated greater overall cytokine activity, both subsets showed all possible combinations of TNF-α, IFN-γ, and IL-10 expression (Figure 3D). Considering the significant activation (Figure 3C) and accumulation (Figure 2, D and E) of CD4^+^ and CD8^+^ EM T cells, it is notable that only a small fraction of both subsets expressed any of these 3 cytokines during infection (Figure 3D), demonstrating the exceptionally tight regulation of these potent immune signaling proteins. Furthermore, IL-10, considered to be a master negative regulator of inflammation (37), was coexpressed with proinflammatory cytokines IFN-γ and to a lesser extent TNF-α (Figure 3D), indicating an additional level of regulation at the single-cell level (40).

Overall, the aforementioned changes in cellular makeup are indicative of a strong Th1 inflammatory immune response (neutrophils, eMs, and Ly6C^+^ Mos) through 3DPI that wanes by 6DPI, giving rise to cells involved in the adaptive immune response (CD4^+^ and CD8^+^ EM T cells) and tissue repair (Ly6C^–^ Mos and iMs). Functional marker expression further supports this narrative, with innate immune cells producing nearly all cytokines at 3DPI, whereas adaptive CD4^+^ and CD8^+^ T cells took over the TNF-α, IFN-γ, and IL-10 production by 6DPI (Figure 3B). Such results are well in agreement with progression of influenza disease (41), validating our CyTOF approach for comprehensive analysis of the immune landscape.

### Influenza protein expression profile reveals differential susceptibility of immune subsets to viral infection and permissiveness to viral replication.

While epithelial cells are considered the primary source of influenza infection and replication, experiments utilizing GFP reporter viruses (42) and single-cell RNA sequencing (43) showed that immune cells can also become infected in vivo. However, identification of susceptible cell types in these experiments was limited to crude immune subsets (e.g.*,* bulk B cell, T cell, NK, and granulocyte populations). Aside from these studies, the relative susceptibility of immune cell types to influenza virus infection or their permissiveness to viral replication in vivo remains largely unexplored. It is unknown whether these 2 properties are affected by host genetic background or aging.

**Figure 4:**
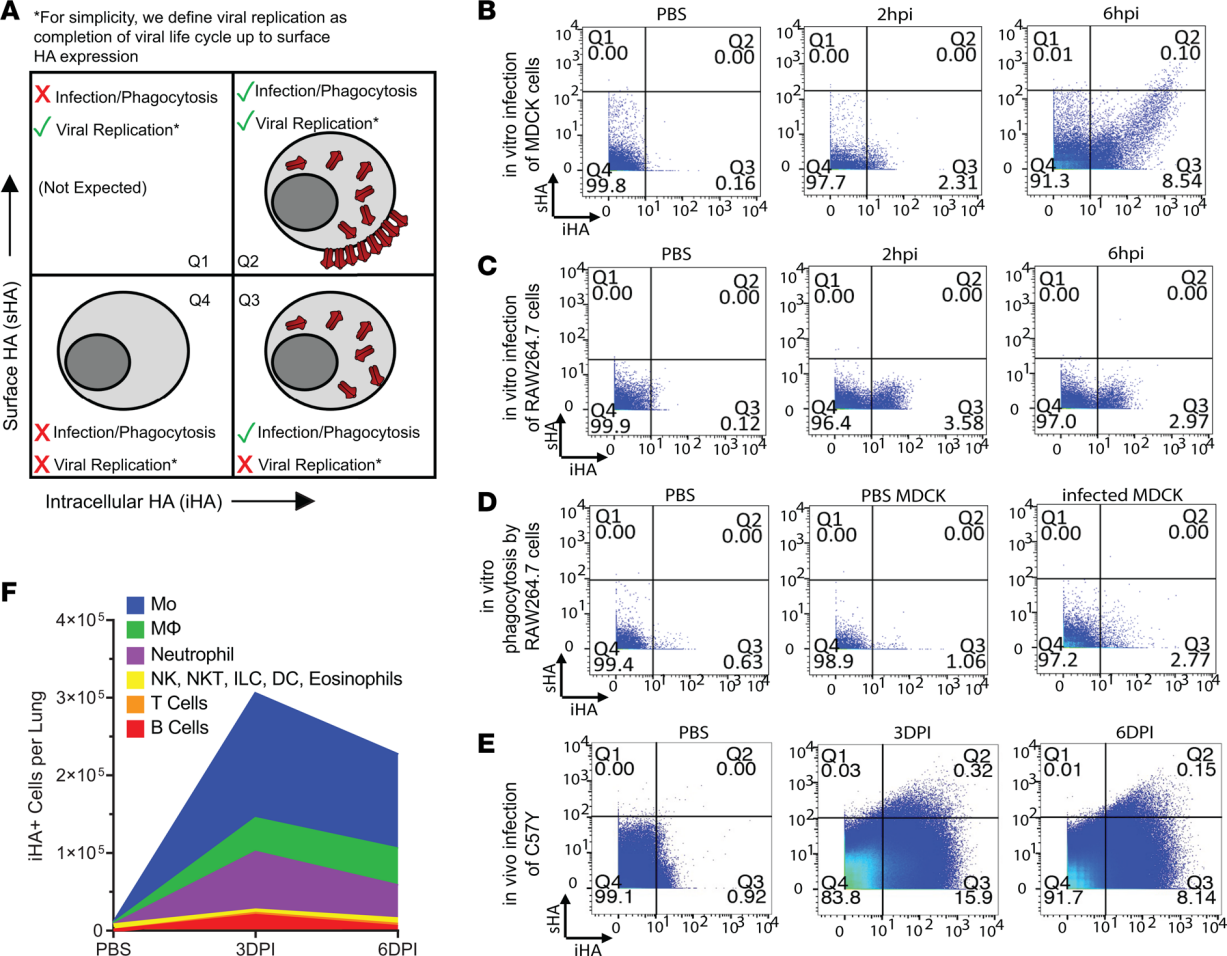
Differential iHA and sHA expression profiles in cells with varying abilities for phagocytosis, susceptibility to influenza infection, and permissiveness to influenza replication. (**A**) Intracellular HA (iHA) signal results from either viral infection or phagocytosis of infected cells, whereas surface HA (sHA) signal can only result from productive viral replication — at least up to the point of viral protein surface expression. In vitro influenza A/PR/8/34 (PR8) infection of (**B**) MDCK and (**C**) RAW264.7 cells at an MOI of 0.05. PBS-treated cells were included as controls. (**D**) In vitro phagocytosis of PBS-treated MDCK cells (middle panel) and PR8-infected MDCK cells (right panel) by RAW264.7 cells. PBS-treated RAW264.7 cells were included as another control. (**E**) Representative biaxial plots of sHA and iHA staining of C57Y CD45^+^ cells. iHA^+^sHA^+^ (Q2) cells actively support viral replication, while iHA^+^sHA^–^ (Q3) cells are either infected or have phagocytosed infected cells but have not succumbed to viral replication up to the point of sHA expression. (**F**) Mean iHA^+^ cell counts per lung by cell type (*n* = 10).

To enable the identification and tracking of infected immune cells, we included 2 channels in the CyTOF antibody panel against the influenza HA protein: one for intracellular staining (iHA) and the other for cell surface staining (sHA). We hypothesized that iHA signal could result from either viral infection or phagocytosis of infected cells, whereas sHA signal could only result from productive viral replication — at least up to the point of viral protein surface expression (Figure 4A).

To test this hypothesis, we first performed in vitro PR8 infection using MDCK (productive PR8 replication; ref. 44) and RAW264.7 (abortive PR8 replication; ref. 45) cells at a low multiplicity of infection (MOI) of 0.05. At 2 hours post infection (2hpi), enough time for viral entry but not for replication (44), iHA was detected in approximately 2.3% of MDCK cells and no sHA signal was observed in any MDCK cells (Figure 4B). As a control, PBS-treated MDCK cells showed no iHA or sHA signal. Therefore, iHA signal can result from infection. At 6hpi, which is enough time for viral replication to occur (44), the iHA level accordingly increased by 10- to 100-fold in some infected MDCK cells as a result of newly synthesized HA protein and approximately 0.10% of MDCK cells with the highest iHA levels became sHA^+^, indicative of successful trafficking of HA protein to the plasma membrane for assembly and budding of new virions. Compared to MDCK, RAW264.7 cells showed a similar level of PR8 endocytosis at 2hpi (~3.6% iHA^+^sHA^–^), but no HA protein synthesis and thus no sHA expression at 6hpi (Figure 4C). This iHA/sHA profile further suggests that the abortive replication of PR8 in RAW264.7 cells is due to an obstruction downstream of virus endocytosis and upstream of protein synthesis, consistent with the findings of a previous study using immunofluorescence microscopy (45). Taken together, these data demonstrate that the sHA signal only results from viral replication. To test whether iHA signal could also result from phagocytosis of infected cells, we next performed in vitro phagocytosis of PR8-infected (MOI = 0.05) MDCK cells using RAW264.7 cells (see Methods and ref. 46). When mixed with PR8-infected MDCK cells, iHA was detected in approximately 2.8% of RAW264.7 cells, but no sHA signal was observed, whereas lower iHA and no sHA signal was detected when PBS-treated or no MDCK cells were added (Figure 4D). Therefore, iHA signal can also result from phagocytosis of infected cells. Together with the observation that very few cells had the unexpected combination of iHA^–^sHA^+^ (Q1) in all conditions tested above (Figure 4, B–D) and in the lung of the PR8-infected C57Y mice (Figure 4E), these data validate our hypothesis and support the use of iHA/sHA staining to enable, for the first time to our knowledge, the dynamic examination of both influenza infection and replication in a wide range of immune cells in vivo. In the data analysis below, we interpret that iHA^+^sHA^+^ (Q2) cells actively support viral replication, while iHA^+^sHA^–^ (Q3) cells are either infected or have phagocytosed infected cells, but have not succumbed to viral replication up to the point of sHA expression (Figure 4A).

**Figure 5:**
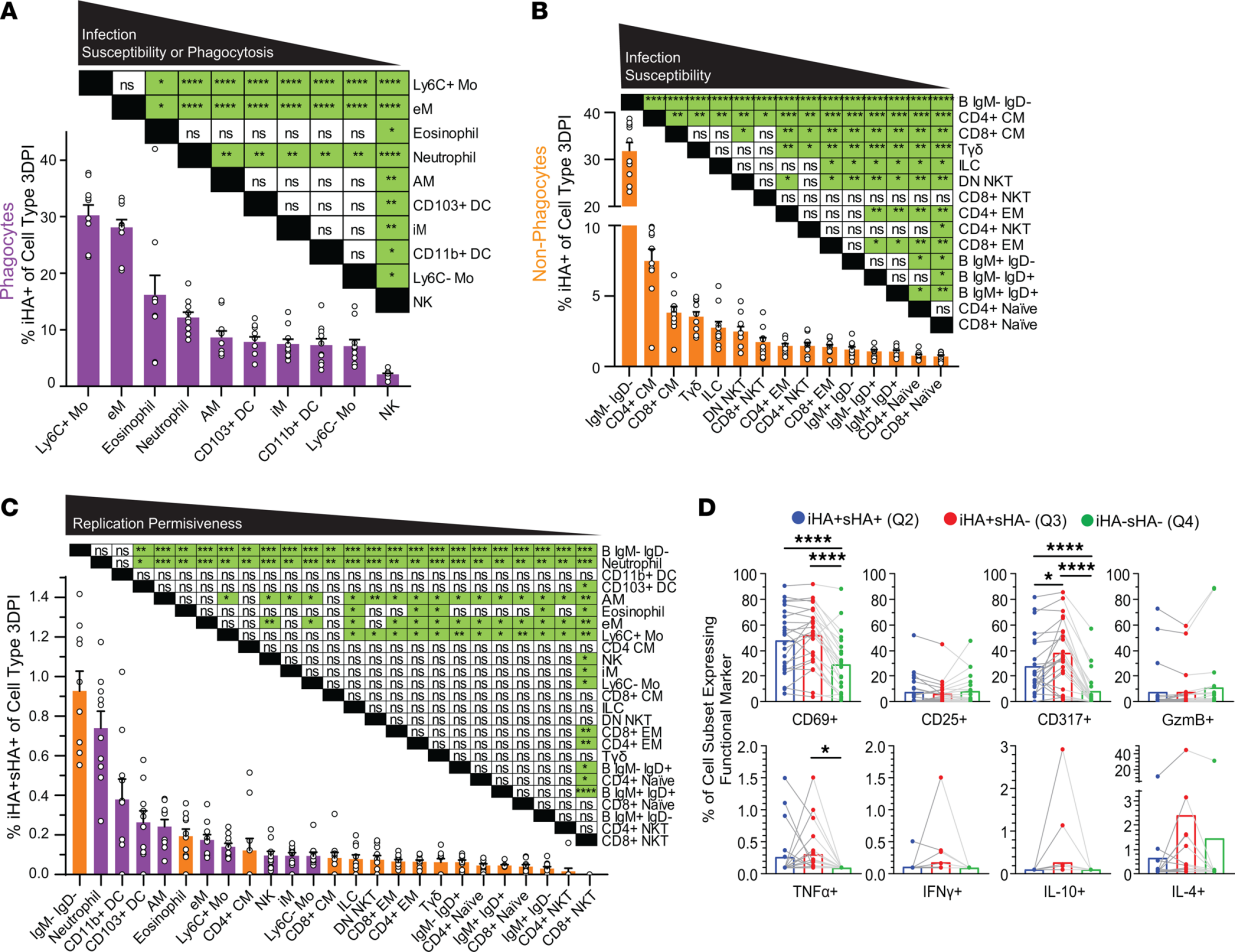
Expression profiling of iHA and sHA in 25 canonical immune cell types. Fraction of intracellular HA–positive (%iHA^+^) cells by cell type for C57Y (**A**) phagocytes and (**B**) nonphagocytes at 3DPI. (**C**) %iHA^+^sHA^+^ by cell type for phagocytes (purple) and nonphagocytes (orange) at 3DPI. (**D**) Mean percentage of cell subsets expressing functional marker for iHA^+^sHA^+^ cells, iHA^+^sHA^–^ cells, and iHA^–^sHA^–^ cells at 3DPI. Each dot represents data for 1 cell type, and lines connect dots of the same cell type. Data represent mean ±SEM, *n* = 10. Statistical comparisons were computed by paired 1-way ANOVA with post hoc Tukey’s pairwise comparisons. *q < 0.10; **q < 0.01; ***q < 0.001; ****q < 0.0001.

Over the course of infection, iHA^+^ immune cell counts indicative of viral infection or phagocytosis peaked (~0.3 million per lung) at 3DPI and decreased by 26% toward 6DPI, with monocytes, macrophages, and neutrophils comprising the vast majority (>90%) of iHA^+^ immune cells (Figure 4F). We next ranked immune cell types by their iHA^+^ fraction (hereafter referred to as %iHA^+^) at 3DPI. The %iHA^+^ of the phagocytic cell types (mostly >7%, Figure 5A) were overall much higher than nonphagocytic cell types (mostly <4%, Figure 5B). For phagocytes, Ly6C^+^ Mos and eMs showed significantly higher %iHA^+^ (*q* < 0.10) than all other phagocytic cell types (Figure 5A); however, it is not clear whether the observed high %iHA^+^ in these cells is due to their high levels of phagocytosis or high infection susceptibility. In contrast with phagocytes, the iHA^+^ signal in nonphagocytic cells primarily results from infection, thus their %iHA^+^ is a quantitative indicator of their susceptibility to influenza infection. With this in mind, the data in Figure 5B suggest that the 15 nonphagocytic immune cell types have differential susceptibility to viral infection, with IgM^–^IgD^–^ B cells and central memory (CM) T cells being the most susceptible (highest %iHA^+^) and naive T cells being the least susceptible (lowest %iHA^+^). Similar findings were also observed at 6DPI (Supplemental Figure 9, A and B). The relative ranking of %iHA^+^ of nonphagocytic cell types at 3DPI is highly correlated with that at 6DPI (Supplemental Figure 9C; Spearman’s δ of 0.93, *P* < 0.0001), suggesting their differential susceptibility to viral infection is cell-type specific and independent of infection time. The mechanism underlying this observed phenomenon is unknown, but is likely related to varying degrees of cellular exposure to influenza virus in the lung.

We next investigated the sHA expression profile to understand which immune cell types support viral replication. Surprisingly, we detected iHA^+^sHA^+^ cells for every immune cell type, demonstrating that all 25 cell types have the ability to support viral replication — at least up to sHA expression (Figure 5C and Supplemental Figure 9D). Furthermore, the %iHA^+^sHA^+^ varies significantly among different immune cell types, indicating that certain cell types are more permissive to viral replication than others. Noticeably, phagocytic cell types showed overall higher permissiveness to viral replication than nonphagocytic cell types (purple vs. orange bars in Figure 5C), which may be a result of their increased interaction with infected cells due to their phagocytic function. The relative ranking of %iHA^+^sHA^+^ remained largely the same at 6DPI (Supplemental Figure 9E; Spearman’s δ of 0.77, *P* < 0.0001), indicating the differential permissiveness to viral replication is also cell-type specific and independent of infection time.

Finally, we investigated whether functional marker expression varies depending on whether cells are (a) supporting viral replication (iHA^+^sHA^+^), (b) infected or have phagocytosed infected cells (iHA^+^sHA^–^), or (c) uninfected (iHA^–^sHA^–^). Most noticeably, the fraction of each immune cell type expressing CD317 and CD69 was substantially increased (*q* < 0.0001) upon infection or phagocytosis (iHA^+^sHA^–^) compared with uninfected cells (iHA^–^sHA^–^) (Figure 5D). However, once the immune cell types succumb to viral replication (iHA^+^sHA^+^), %CD317^+^ decreases for the vast majority of the 25 immune cell types (*q* < 0.10) (Figure 5D). These data suggest that cells are more susceptible to viral replication when the expression of these markers is low. Supporting this, CD317 (i.e., tetherin) is an IFN-inducible protein known to play a critical role in preventing the release of enveloped viruses from the cell surface (47).

Taken together, these results demonstrate that analyzing the iHA and sHA expression profile can uncover knowledge on viral infection and replication in different immune cell types. Our data suggest that immune cell types possess varying in vivo susceptibility to viral infection (Figure 5B) and permissiveness to viral replication (Figure 5C). Furthermore, we identified changes in functional marker signatures that depended on the viral status in immune cells (Figure 5D). Compared with previous studies on profiling the dynamic changes in cell populations and their functional marker expression, the incorporation of viral proteins in CyTOF analysis in this study provides insight into in vivo virus-specific behavior.

### Effect of host genotype on immune cellular makeup and cytokine environment in the lung.

**Figure 6:**
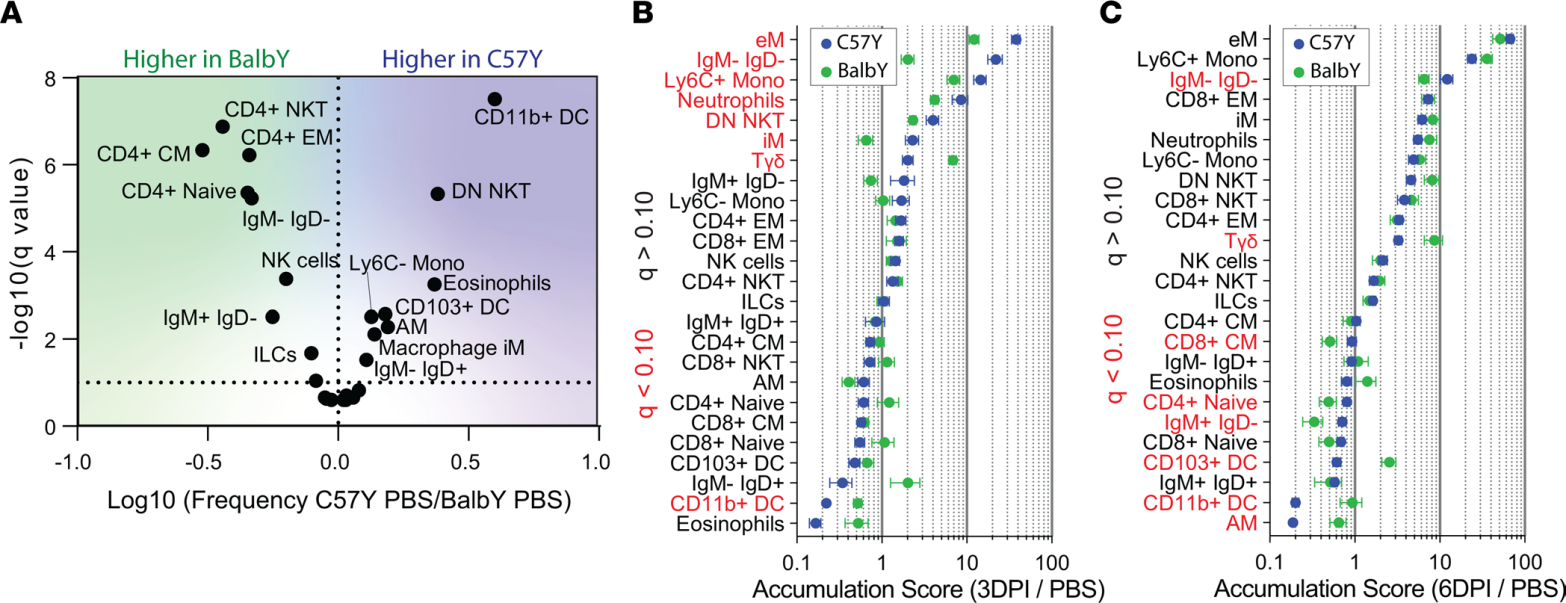
Effect of genotype on immune cell makeup in response to influenza virus infection. (**A**) Volcano plot indicates the fold difference in cell type frequency between C57Y and BalbY mice treated with PBS (*n* = 10). Accumulation scores of C57Y and BalbY mice at (**B**) 3DPI and at (**C**) 6DPI. C57Y data in **B** and **C** are also shown in Figure 2E. Accumulation scores were calculated as the mean fold change in absolute cell counts for each cell type 3DPI or 6DPI compared with PBS. Cell subtypes are ordered based on C57Y ranking. Significantly different subsets are indicated in red. Data represent mean ± SEM, *n* = 10. Statistical comparisons were computed by 2-sided Student’s *t* test with FDR = 10%.

We next applied iGATE to study the effects of host genotype on the immune response to influenza infection in the mouse lung. Our challenge data (Supplemental Figure 1) and prior studies have demonstrated that BALB/c mice are more resistant to H1N1 influenza infection than C57BL/6 mice (27–29). Thus, these 2 genotypes represent an ideal model to explore influenza resistance due to host genetic background. To this end, we first compared the basal level difference in cell type frequencies between the resting state of BalbY and C57Y mice treated with PBS (Figure 6A). Most noticeably, BalbY mice had significantly higher frequency of CD4^+^ T cell subsets than C57Y mice. While no direct comparison of C57BL/6 and BALB/c cellular subsets in the lung exists in the literature to our knowledge, the increased level of CD4^+^ T cells in BALB/c mice has been corroborated in PBMCs and splenocytes (48).

**Figure 7:**
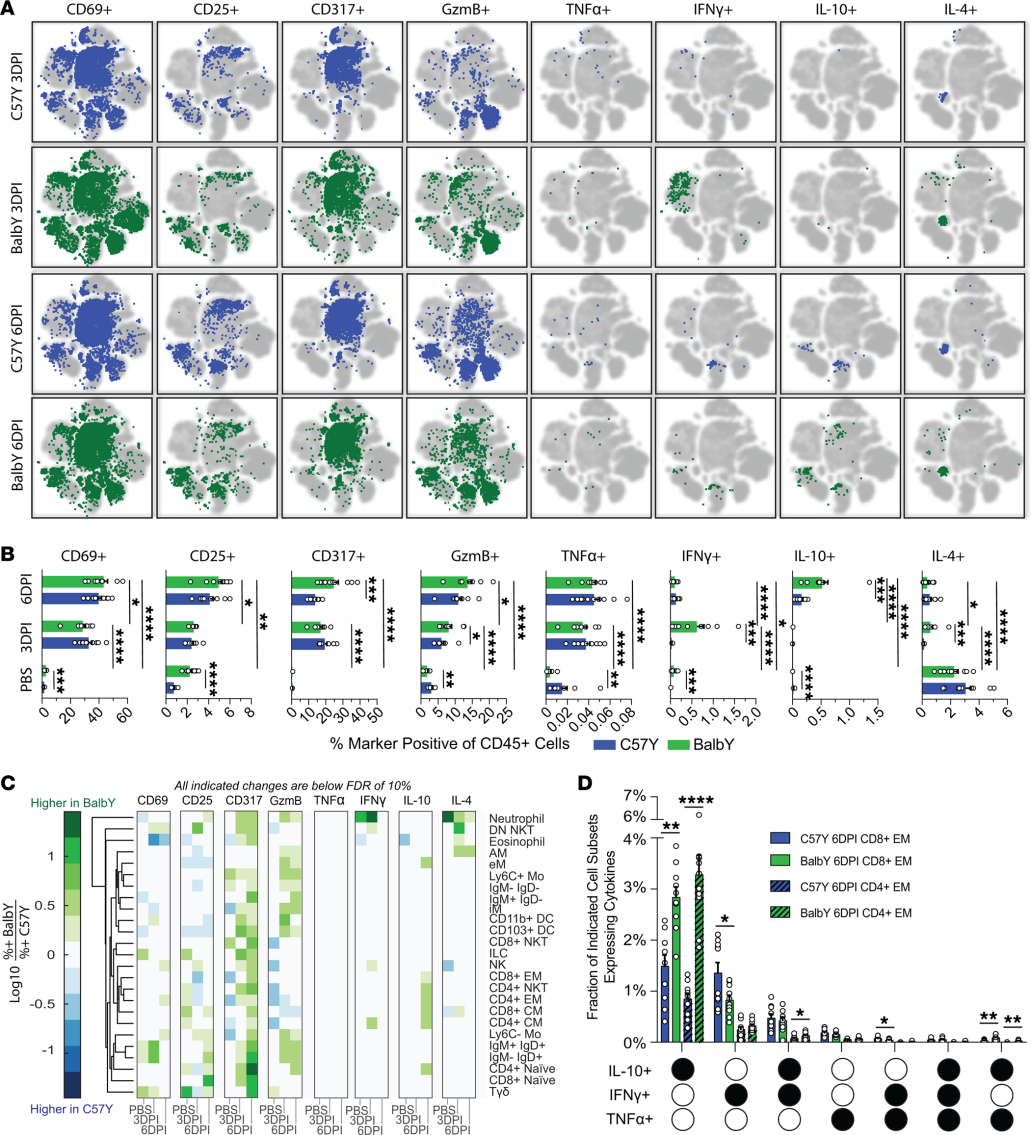
Effect of genotype on phenotype and function of immune cell types in response to influenza virus infection. (**A**) Comparison of functional marker positive cells on the viSNE map. Refer to Figure 2A for cell type distribution on viSNE map. (**B**) Bar charts comparing marker positive cell frequency out of total CD45^+^ cells between C57Y (blue) and BalbY (green). C57Y data are also shown in Figure 3A. (**C**) Log(fold change) of the percentage of marker expression for each cell type. (**D**) Comparison of cytokine coproduction by CD4^+^ and CD8^+^ EM T cells at 6DPI. C57Y data are also shown in Figure 3D. Data represent mean ± SEM, *n* = 10. Statistical comparisons were computed by 2-sided Student’s *t* test with FDR = 10%. *q < 0.10; **q < 0.01; ***q < 0.001; ****q < 0.0001.

Upon influenza virus infection, the 25 immune cell types exhibited dynamic, differential accumulation and depletion in the lungs of the 2 genotypes at 3DPI (Figure 6B) and 6DPI (Figure 6C). Compared with C57Y, BalbY were less responsive during early stages of influenza infection, as indicated by significantly lower-magnitude changes in cell counts of both accumulating and depleting cell types in the lung at 3DPI (Figure 6B). By 6DPI, the accumulation of infiltrating immune cell types, namely eMs, Ly6C^+^ Mos, neutrophils, and iMs in C57Y and BalbY reached similar levels (Figure 6C). These results suggest a less aggressive inflammatory response in BalbY. Despite the reduced accumulation of inflammatory cell types at 3DPI (Figure 6B), BalbY had significantly more immune cells producing inflammatory IFN-γ and cytotoxic GzmB at 3DPI than C57Y (Figure 7, A and B). This strong early IFN-γ response in BalbY likely contributed to a significantly (*q* < 0.001) greater frequency of cells upregulating CD317 (Figure 7B) at 6DPI. Interestingly, BalbY also mounted a much stronger antiinflammatory IL-10 response at 6DPI than C57Y to more rapidly contain the IFN-γ response (Figure 7, A and B).

To determine cell-type-specific contributions to these bulk differences in functional marker signatures, we compared marker-positive fractions for each immune cell type (Figure 7C). The greater IFN-γ production in BalbY at 3DPI was attributed primarily (~92%) to neutrophils (Supplemental Figure 10). Neutrophil IFN-γ production has received little attention, but may be beneficial given the broad-spectrum antiviral properties of IFN-γ (49–52). In contrast, the upregulation of CD317 was observed in the majority of the 25 immune cell types (Figure 7C), indicating enhanced viral sensing and upregulation of IFN-inducible proteins that may be important for influenza resistance. The increased IL-10 production in BalbY at 6DPI was also observed in several immune cell types (Figure 7C), with CD4^+^ and CD8^+^ EM T cells representing the major (~80%) cellular sources of IL-10 (Supplemental Figure 10). Analysis of IL-10, IFN-γ, and TNF-α combinatorial production by CD4^+^ and CD8^+^ EM T cells revealed significantly more IL-10 single-positive cells in BalbY compared with C57Y at 6DPI (*q* < 0.01, Figure 7D), demonstrating a more regulatory environment in the lung of BalbY mice.

### Effect of host genotype on immune cell susceptibility to influenza infection and permissiveness to influenza replication.

**Figure 8:**
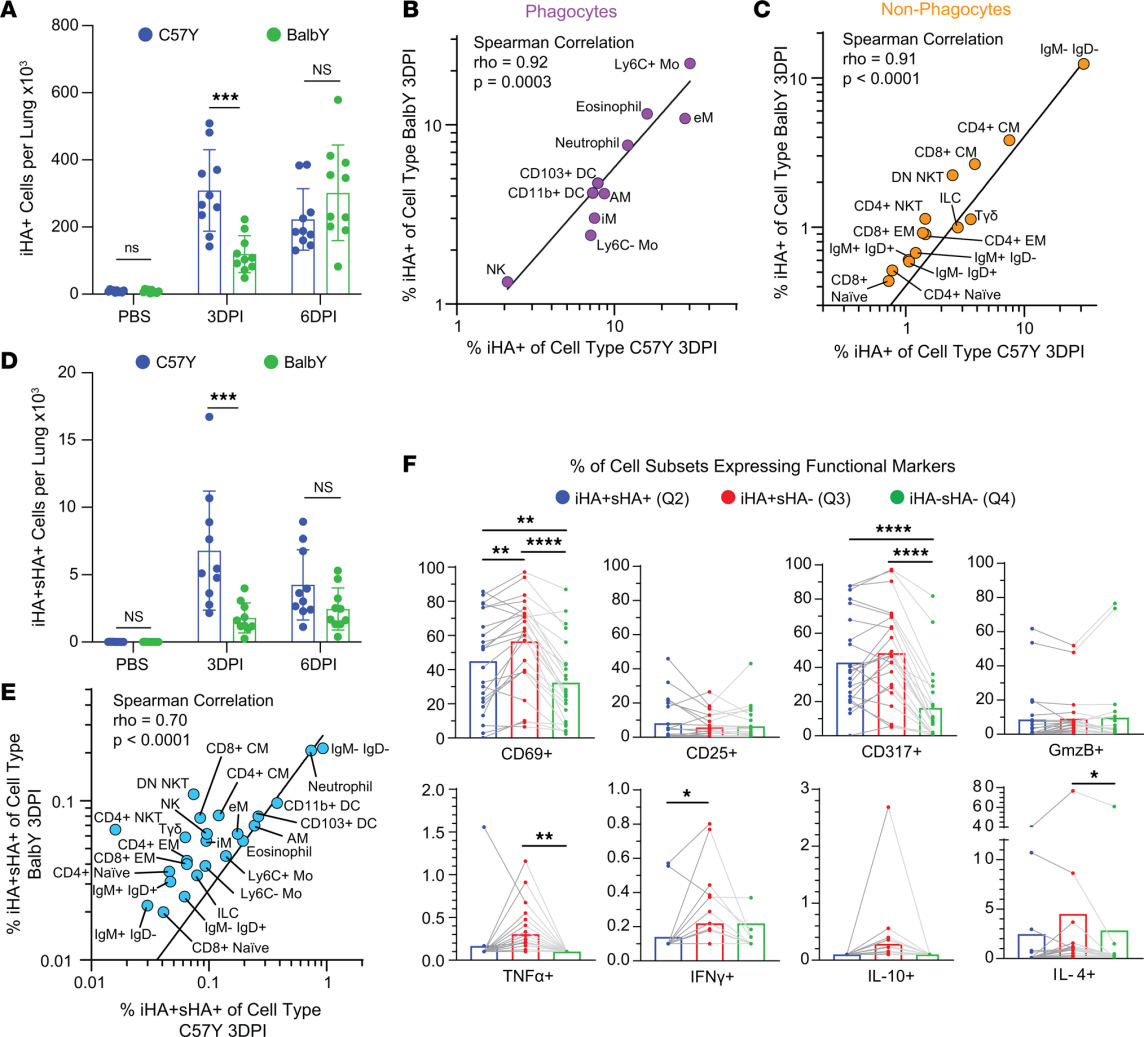
Effect of host genotype on iHA and sHA expression in immune cell types. (**A**) Comparison of iHA^+^ cell counts between C57Y and BalbY mice (*n* = 10). Comparison of mean %iHA^+^ for each cell type between C57Y and BalbY mice for (**B**) phagocytes and (**C**) nonphagocytes. (**D**) Comparison of iHA^+^sHA^+^ cell counts between C57Y and BalbY mice. (**E**) Comparison of mean %iHA^+^sHA^+^ of all immune cell types between C57Y and BalbY. (**F**) Mean percentage of BalbY cell subsets expressing functional markers for iHA^+^sHA^+^ cells, iHA^+^sHA^–^ cells, and iHA^–^sHA^–^ cells at 3DPI. Each data point represents 1 immune cell type and lines connect cells of the same type. Statistical comparisons in **B**, **C**, and **E** were computed by Spearman’s correlation, and a linear regression line is also shown. All data represent mean ± SEM, *n* = 10; statistical comparisons in **A** and **D** were computed by 2-sided Student’s *t* test with FDR of 10%; statistical comparisons in **F** were computed by paired 1-way ANOVA with post hoc Tukey’s pairwise comparisons. *q < 0.10; **q < 0.01; ***q < 0.001; ****q < 0.0001.

Compared with C57Y, BalbY demonstrated both a significantly lower number (*q* < 0.001, Figure 8A) and frequency (*q* < 0.0001, Supplemental Figure 11A) of iHA^+^ immune cells at 3DPI before reaching similar levels by 6DPI. These results suggest an overall reduced viral activity (infection and phagocytosis) in BalbY compared with C57Y at the early stage of viral infection, corroborated by the stronger antiviral cytokine (IFN-γ and GzmB) environment observed at 3DPI in the BalbY lung (Figure 7, A and B).

Interestingly, the relative ranking of %iHA^+^ values for the 25 immune cell types at 3DPI were highly correlated between BalbY and C57Y for both phagocytic (Spearman’s δ of 0.92, *P* = 0.0003) and nonphagocytic cell types (Spearman’s δ of 0.91, *P* < 0.0001) (Figure 8, B and C). Similar results were obtained at 6DPI (Supplemental Figure 11, B and C). These results suggest that host genotype does not substantially alter the relative phagocytic ability or infection susceptibility of the 25 immune cell types.

Next, we investigated the effect of host genetic background on viral replication in immune cell types. Compared with C57Y, BalbY demonstrated a significantly lower number and frequency of iHA^+^sHA^+^ immune cells over the course of infection (Figure 8D and Supplemental Figure 11D). This result indicates an overall reduced viral replication in BalbY, which is an expected outcome of the reduced viral activity as described in Figure 8A. Spearman’s correlation analysis also revealed statistically correlated ranking of %iHA^+^sHA^+^ values for the 25 immune cell types between BalbY and C57Y over the course of infection (Figure 8E and Supplemental Figure 11E), suggesting that genotype does not substantially alter the relative permissiveness of immune cell types to viral replication.

To further explore potential mechanisms of the observed overall higher resistance of BalbY to viral infection/phagocytosis (Figure 8A) and replication (Figure 8D) at the individual immune cell-type level, we investigated changes in functional marker expression in BalbY cells in Q2 (iHA^+^sHA^+^), Q3 (iHA^+^sHA^–^), and Q4 (iHA^–^sHA^–^) for each cell type (Figure 8F). Overall, changes in functional marker expression were similar to those in C57Y, as described in Figure 5D. In particular, immune cell types that succumbed to viral replication (iHA^+^sHA^+^) had an overall lower fraction of cells expressing functional markers, including CD317, CD69, and IFN-γ compared with iHA^+^sHA^–^ cells. In addition, the fraction of cell types in Q2 and Q3 expressing functional markers did not greatly change with genotype (Supplemental Figure 11, F and G). While these results further reinforce the finding that viral status alters cellular functional marker expression in both BalbY and C57Y, they provide no clear evidence that this change at the individual immune cell–type level may explain the observed higher resistance of BalbY mice to influenza infection.

### Aging alters the makeup and cytokine profile of immune cells, but not their relative susceptibility to influenza infection or permissiveness to influenza replication.

**Figure 9:**
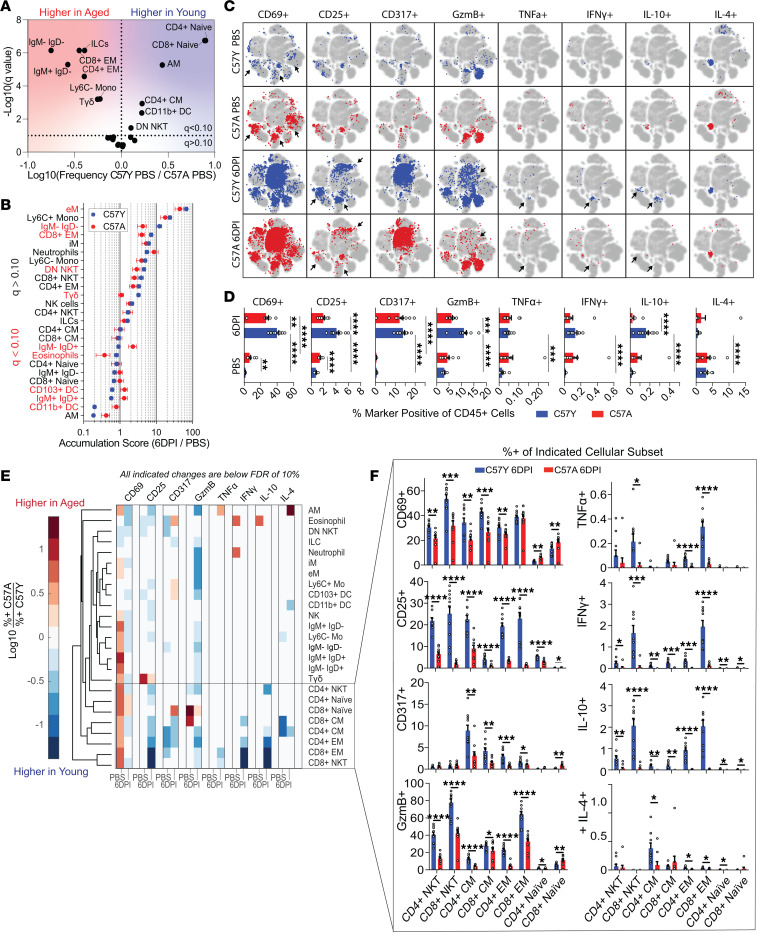
Effect of aging on immune response to influenza virus infection. (**A**) Volcano plot indicates the fold difference in cell type frequency between C57Y and C57A mice treated with PBS (*n* = 10). (**B**) Comparison of accumulation scores at 6DPI between C57Y (*n* = 10) and C57A (*n* = 9) mice. Immune cell types are ordered based on C57Y. C57Y data are also shown in Figure 2E. Significantly different subsets are indicated in red. (**C**) Comparison of functional marker–positive cells on viSNE. Refer to Figure 2A for cell type distribution on viSNE map. C57Y plots are also shown in Figure 7A. (**D**) Bar charts comparing C57Y and C57A marker frequency out of total CD45^+^ cells. C57Y data are also shown in Figure 3A. (**E**) Log(fold change) of marker expression frequency for each cell type at 6DPI. (**F**) Functional marker expression in NKT and T cell subsets. Data represent mean ± SEM. Statistical comparisons were computed by 2-sided Student’s *t* test with FDR = 10%. *q < 0.10; **q < 0.01; ***q < 0.001; ****q < 0.0001.

We next investigated how aging, a well-known host factor associated with greater influenza disease severity, affects the immune responses by comparing the immune landscape of C57Y mice to that of C57A. To understand basal level differences between young (2 month) and aged (18 month) mice, we compared cell type frequencies between the 2 PBS-treated groups (Figure 9A). Thirteen of the 25 immune cell types exhibited significant changes in frequency upon aging. The greatest variation resided in the T cell compartment, where C57A had significantly greater frequencies of CD4^+^ and CD8^+^ EM T cells, while C57Y had significantly more naive T cells, an expected outcome that has been well reported (48). In addition, we identified greater AM frequencies in C57Y, which has also been observed by others (53, 54).

Following influenza virus infection, the accumulation scores of all immune cell types at 6DPI were compared between C57Y and C57A, with significantly different subsets (*q* < 0.10) highlighted in red (Figure 9B). While the overall trends are similar between the 2 groups, C57A generally displayed less responsiveness, with only marginal increases in immune cell types with positive accumulation scores and much smaller decreases in those with negative accumulation scores compared with C57Y. In particular, C57A had significantly lower accumulation of CD8^+^ EM T cells and Tγδ cells, the former being a critical cell type known to directly participate in the killing of virus-infected cells (55). In addition, C57A showed significantly greater depletion of eosinophils, but less depletion of CD11b^+^ DCs. While the mechanisms underlying this observation are unknown, it has been reported that DCs in aged mice are defective in their migration to draining lymph nodes (56). Taken together, these results suggest that aging leads to an altered immune cell makeup in the lung in response to influenza infection, possibly through altered trafficking and/or defective cellular proliferation.

To gain insights into how aging affects functional marker expression in immune cell types, marker-positive cells were overlaid on the viSNE map (Figure 9C). While C57A had similar marker expression profiles compared to C57Y, some notable differences were observed (arrowed populations, Figure 9C). Specifically, PBS-treated C57A mice had increased frequencies of immune cells expressing CD69, CD25, TNF-α, IFN-γ, and IL-10 (Figure 9D), consistent with reports of elevated basal level activation and inflammation associated with aging (i.e., inflammaging) (57). However, this basal level of activity in C57A did not translate into efficient activation during infection. On the contrary, C57A at 6DPI actually showed reduced activation, with lower frequencies of CD69^+^ and CD25^+^ immune cells as well as lower expression of IFN-γ, GzmB, and IL-10 cytokines compared with C57Y. The reduction in IFN-γ and IL-10 in aged mice was consistent with their decreased levels measured in BAL (Supplemental Figure 8). To further determine the affected immune cell types, we compared the marker-positive fraction of each immune cell type in C57A to that in C57Y (Figure 9E). Hierarchical clustering clearly separated T cells and NKT cells of both CD4^+^ and CD8^+^ lineages (boxed in Figure 9E) from other cell types, indicating that these subsets are particularly affected by aging. Indeed, aged mice exhibited severe functional defects in response to influenza infection, showing significantly reduced activation and cytokine production at 6DPI compared with their young counterparts (Figure 9F).

Finally, we investigated the HA expression profile at 6DPI in C57A compared to that in C57Y. Overall, no significant difference was observed in the total number of iHA^+^ immune cells (Supplemental Figure 12A), the relative phagocytic ability or susceptibility of individual cell types to influenza infection (Supplemental Figure 12, B and C), the total number of iHA^+^sHA^+^ immune cells (Supplemental Figure 12D), or the relative permissiveness of individual cell types to influenza replication (Supplemental Figure 12E). These results indicate that the relative susceptibility of immune cell types to viral infection and permissiveness to viral replication are largely unaffected by aging.

## Discussion

With the 40-marker CyTOF antibody panel and iGATE developed in this study, 29 million CD45^+^ live single cells were rapidly and accurately classified in an automated process into 25 canonical immune cell types, improving the interpretability of the immune landscape during early-stage influenza infection in mouse lung. Specifically, in C57Y mice, we detailed the early inflammatory response at 3DPI followed by the initiation of the adaptive arm by 6DPI (Figures 2 and 3). In addition to expected changes in cellular frequencies, we observed unanticipated changes in immune cell types not typically investigated in the context of viral infections, including Tγδ and eosinophils (Figure 2, D and E), suggesting they play underappreciated roles in antiviral immunity. While research is limited, a few studies have shed some light on their potential antiviral mechanisms. Tγδ cells were shown to participate in both innate and adaptive influenza immune responses via their diverse abilities to be innately activated by influenza HA (58), present influenza viral peptides to αβT cells via MHC-II (59), and kill virus-infected cells (60). For eosinophils, it has been demonstrated that mouse eosinophils become activated in the presence of influenza virus (61), secrete RNases that decrease viral infectivity in vitro (62), undergo piecemeal degranulation upon viral exposure (61), and present influenza antigen via MHC-I and MHC-II that results in activation and proliferation of CD8^+^ and CD4^+^ T cells (61, 63). Therefore, Tγδ cells and eosinophils represent promising therapeutic targets for influenza treatment. Indeed, a high-fat ketogenic diet led to increased Tγδ cell counts in mouse lungs, improving H1N1 protection (64), while transfer of lung eosinophils from allergen-sensitized mice into H1N1-infected mice reduced viral burden and morbidity (61).

Our ability to profile the iHA and sHA expression enabled a comprehensive evaluation of 2 properties of 25 immune cell types in vivo: susceptibility to influenza infection and permissiveness to influenza replication. Note that this study only considered immune cells, excluding nonimmune cells. We demonstrate for the first time to our knowledge that, not only are all 25 immune cell types susceptible to influenza infection, but they are also permissive to influenza replication in vivo — at least up to the point of surface HA expression (Figure 5C and Supplemental Figure 9D). We further showed that both properties are cell-type specific and conserved over the course of infection (Supplemental Figure 9, C and E) and across mice with differing genotypes (Figure 8, B, C, and E) and ages (Supplemental Figure 12, B, C, and E). Interestingly, CD4^+^ and CD8^+^ CM T cells were among the most susceptible to infection (high %iHA^+^) of all nonphagocytic cells, while naive CD4^+^ and CD8^+^ T cells were the least susceptible (low %iHA^+^, Figure 5B and Supplemental Figure 9B). This result demonstrates intracompartmental (T cell subsets) heterogeneity in susceptibility to influenza infection. Together with the observation that phagocytes were generally more permissive to influenza replication than nonphagocytes (Figure 5C), these data suggest that both properties generally correlate with the expected exposure of cell types to influenza virus in vivo while carrying out their functions. For example, phagocytes engulf infected cells and memory T cells home to sites of inflammation, but naive cells largely bypass tissues en route to draining lymph nodes (65). B cells, in particular the IgM^–^IgD^–^ subset, take up antigens through endocytosis or phagocytosis (66–68).

The ability to simultaneously detect iHA and sHA expression on a single cell further enabled us to differentiate cells into 3 categories (Figure 4, A–E): (a) uninfected (iHA^–^sHA^–^), (b) cells that are infected or have phagocytosed infected cells but have not succumbed to viral replication up to the point of sHA expression (iHA^+^sHA^–^), and (c) cells that support viral replication (iHA^+^sHA^+^). Our data provide insight into the effect of viral status on functional marker expression in immune cells. Most notably, across 25 immune cell types, we noted a significant decrease in %CD317^+^ in the iHA^+^sHA^+^ subpopulation compared with the iHA^+^sHA^–^ subpopulation (Figure 5D and Figure 8F). Given that CD317 prevents the release of enveloped viruses from the cell surface (47), these results suggest that the downregulation of CD317 — and likely other type I IFN–inducible proteins as well — is important for supporting influenza replication in immune cells in vivo. Indeed, several type I IFN–inducible genes have direct antiviral properties (i.e., viperin) (69), and influenza and other viruses have evolved mechanisms to actively suppress the type I IFN response to promote viral replication (51). Further supporting the importance of the type I IFN response in fighting influenza infection, BalbY mice with the best survival showed increased CD317 expression in a majority of the 25 immune cell types when compared with C57Y (Figure 7C). In contrast, C57A mice with the worst survival showed similar CD317 expression to that of C57Y in the majority of the 25 immune cell types (Figure 9E). This result suggests that the reduction in survival associated with aging is not due to the cellular mechanisms for upregulating CD317, but rather due to other deficiencies.

Indeed, we observed several other defects in the C57A immune response that may explain their reduced survival. Compared with C57Y, C57A mice were systematically less responsive to influenza infection, displaying lesser-magnitude changes in immune cell populations (Figure 9B) as well as reduced cellular activation in the lung (Figure 9D). In fact, the overall frequency of cytokine-positive immune cells was largely unchanged at 6DPI in C57A compared to the PBS-treated C57A (Figure 9D). Most notably, C57A displayed severe defects in the T cell and NKT cell compartments. In particular, CD8^+^ EM T cells had approximately 2-fold lower accumulation (*q* < 0.10) compared with C57Y at 6DPI (Figure 9B) and had markedly reduced or nearly undetectable levels of GzmB^+^, TNF-α^+^, IFN-γ^+^, and IL-10^+^ T cells and NKT cells (Figure 9F). Due to the critical roles of effector T cells and these cytokines in combatting viral infection, aged mice are deficient in a key component of antiviral immunity. Thus, we conclude that decreased survival due to aging is associated with marked deficiencies in immune cell accumulation, cellular activation, and cytokine production. While it is well known that aging leads to reduced functionality in effector T cells (70), it is noteworthy that our in-depth analysis identified T cell defects as the most striking deficiency in the early immune response of aged mice.

Regarding host genotype, we identified several factors contributing to the enhanced survival of BalbY over C57Y mice. Aside from the aforementioned increased frequencies of Tγδ, eosinophils, and CD317^+^ immune cells in BalbY, BalbY mice had a more measured inflammatory response, with less infiltration at 3DPI (Figure 6B) and increased levels of antiinflammatory IL-10 at 6DPI (Figure 7, A and B). While the mechanism of increased IL-10 production in BalbY is unknown, there is evidence in the literature that a greater type I IFN response leads to increased production of IL-10 (71). This is in agreement with the higher frequencies of CD317^+^ immune cells observed in BalbY (Figure 7C) compared with C57Y. Despite the importance of IL-10 in anti-influenza immunity suggested by this study, a review of the literature reveals that the IL-10 production in these 2 mouse strains is stimuli dependent (71, 72) and can lead to opposing outcomes in different diseases (73). Thus, we conclude that an appropriately regulated immune response, rather than high levels of the IL-10 cytokine itself, is essential to achieving positive outcomes.

The results of this study have several implications. Our data suggest the perils of both overreactive (in the case of C57Y) and underreactive (in the case of C57A) inflammatory responses during early stages of influenza infection, suggesting the need for different vaccine and therapeutic approaches for different age groups. For example, the deficiencies we observed in C57A T cell functional marker expression (Figure 9F) and the lower accumulation score for CD8^+^ EM T cells in C57A compared with C57Y (Figure 9B) indicate that vaccines that induce T cell responses might be particularly advantageous to the elderly. In the case of genetic background, we clearly demonstrate that genotype affects cellular infiltration and cytokine environment, ultimately leading to differences in survival that could also inform vaccine design. Since higher levels of functional markers associated with IFN-γ expression (IL-10, CD317) are seen in BalbY with the best survival, it could be beneficial to design influenza vaccines that target a Th1 immune response, particularly in individuals who lack robust Th1 immunity. These data highlight the need to test vaccines broadly across diverse genetic backgrounds for better efficacy evaluation.

Beyond providing insights into the effects of aging and genetics on the immune response to influenza infection, our experimental design and iGATE establish a framework for several future directions. Our approach can be directly applied to investigate how other host factors like comorbidities, nutrition, and microbiota impact the influenza immune landscape (10). Most importantly, the techniques developed in this work will prove useful for evaluating vaccines. For example, iGATE can be applied to identify user-defined cell types in vivo that are interacting with the vaccine immunogen and how they are responding in terms of cytokine and functional marker expression at the single-cell level. This information, in particular, is difficult to obtain using other high-dimensional analysis techniques, such as RNA sequencing. Ultimately, such data can help elucidate the protective mechanisms of vaccines, enabling a more predictive approach to vaccine design.

## Methods

### Sex as a biological variable.

Our study exclusively examined female mice as a case study for developing and applying iGATE. Further investigation is required to evaluate whether the findings are relevant for male mice.

### Animal handling, infection, and sample collection.

Female C57BL/6 (2 or 18 months old) and BALB/c mice (2 months old) were obtained from The Jackson Laboratory and 10 mice were used per treatment group. For survival study, mice were anesthetized with isoflurane and instilled intranasally with 2.0 × 10^4^ PFU of influenza virus PR8 (ATCC, VR-1469). For CyTOF experiments, mice were infected as described above with a dose of 1.2 × 10^4^ PFU, corresponding to approximately the LD_50_ of BalbY mice (data not shown). Following infection, mice were monitored daily for health status. One aged mouse that showed contrary weight response after infection was omitted from analysis. At indicated time points, the mice were euthanized, their lungs perfused and harvested, and stored in PBS with Golgi block consisting of brefeldin A (BioLegend) and monensin (BioLegend). These lungs were then minced, digested, and processed into single-cell suspension using 1 mg/mL Collagenase D (Roche), 1 mg/mL DNase I (Roche), and Golgi block. Live cells were further isolated over Ficoll-Paque (Cytiva) and resuspended in PBS with Golgi block prior to CyTOF analysis.

### CyTOF antibody conjugation, staining, and acquisition.

CyTOF antibody conjugation, staining, and data acquisition were performed as previously described (74), with the following modifications: the antibody cocktail for intracellular staining was prepared in the eBioscience permeabilization buffer (10×, 00-8333-56); the samples were acquired on a CyTOF Helios system (Fluidigm Corp.) at approximately 300–400 events/s; and the mouse samples were acquired on the Helios in batches. Within each batch, a control sample of influenza-infected C57Y mouse lung homogenate was included. No substantial variation in identified cell populations in the control sample was observed in any batch (Supplemental Figure 13A and Supplemental Table 2).

### Data analysis.

Following acquisition, signal spillover correction was applied, using measured metal impurities as described previously (75). The probabilistic SVM classifier was trained and validated using the iGATE_train software and deployed on the 29-million-cell data set using the iGATE_predict software. The numerical values of the threshold for the functional marker expression in classified immune subsets were determined by manual gating (Figure 3A) and then applied to all cells in the iGATE analysis. After iGATE had classified all cells, no substantial biological variation in cell populations was observed between mice in the same treatment group (Supplemental Figure 13B). Event counts for all the populations referenced in this study can be found in Supplemental Tables 3–11.

### In vitro infection and in vitro phagocytosis.

MDCK (ATCC, CCL-34) and RAW264.7 (ATCC, TIB-71) cells were infected with PR8 at an MOI of 0.0 (i.e., PBS) or 0.05 for 2 and 6 hours. Cells were trypsinized, washed, and stained to detect iHA and sHA using the CyTOF protocol described above. For in vitro phagocytosis, MDCK cells were infected with PR8 at an MOI of 0.0 (i.e., PBS) or 0.05 for 18 hours. After infection, the MDCK cells were trypsinized, washed, and fed to RAW264.7 cells at a ratio of 1:2. The phagocytosis was carried out in an incubator at 37°C with 5% CO_2_ for 90 minutes. Following phagocytosis, the cells were washed, trypsinized, scraped from the wells, and stained to detect iHA and sHA using the CyTOF protocol described above.

### Statistics.

Statistical analyses were done using Mathworks MatLab or GraphPad Prism v10. Variables were analyzed by either 2-sided Student’s *t* test or 1-way ANOVA as specified in figure legends. For single tests, a *P* value of less than 0.05 was considered statistically significant. For multiple tests, the Benjamini-Hochberg correction was used, and a *q* value of less than 0.10 was considered statistically significant. Correlation analysis was performed using Spearman’s correlation.

### Study approval.

Mice were housed in an AAALAC-accredited facility, in compliance with the Public Health Service Policy on Humane Care and Use of Laboratory Animals and the NIH *Guide for the Care and Use of Laboratory Animals* (76). All procedures were approved by the IACUC at the University of Michigan.

### Data availability.

iGATE software, documentation, and test data set are available for noncommercial use only at https://github.com/UmichWenLab/iGATE Commercial use will require a license — please contact feiwenum@umich.edu for further information. Values for all data points in graphs are reported in the Supporting Data Values file. Any additional information is available from the corresponding author upon reasonable request.

## Author contributions

FW conceptualized and supervised the project. AJZ and SMR performed the experiments. SR, LFB, and FW developed the software. BDH, FW, AJZ, LFB, and HNN wrote the manuscript. BDH, LFB, FW, AJZ, SR, HJ, and SBR performed the data analysis. AO, DRG, and JC contributed to data interpretation and discussion. As co–first authors, BDH is listed first for performing most of the data analysis and AJZ second for performing most of the experiments. All authors reviewed, commented on, and approved the final version of the manuscript.

## Supplementary Material

Supplemental data

Supplemental tables 2-11

Supporting data values

## Figures and Tables

**Table 1:**
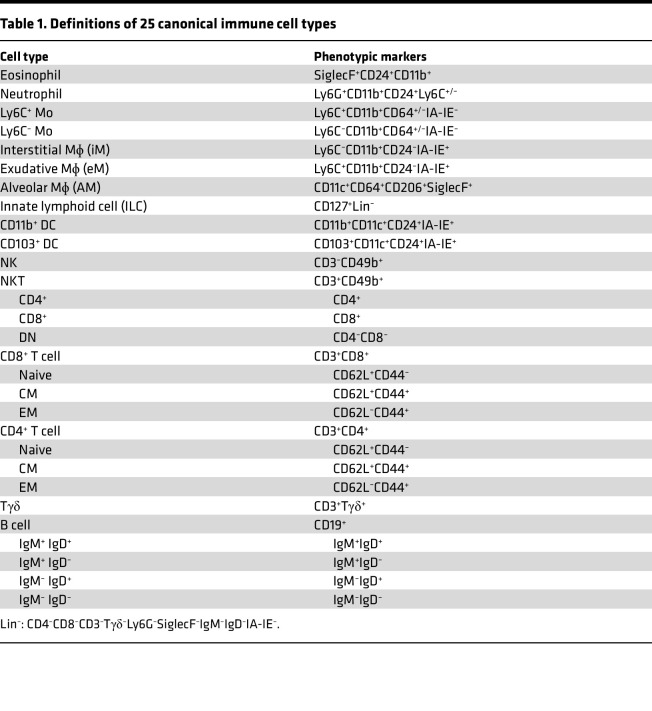
Definitions of 25 canonical immune cell types

## Version 1 05/30/2024

In-Press Preview

## Version 2 06/24/2024

Electronic publication

## References

[1] Wang X (2020). Global burden of respiratory infections associated with seasonal influenza in children under 5 years in 2018: a systematic review and modelling study. Lancet Glob Health.

[2] https://www.cdc.gov/flu/vaccines-work/effectiveness-studies.htm.

[3] Erbelding EJ (2018). A universal influenza vaccine: the strategic plan for the National Institute of Allergy and Infectious Diseases. J Infect Dis.

[4] Zak AJ (2019). Enhancing the yield and quality of influenza virus-like particles (VLPs) produced in insect cells by inhibiting cytopathic effects of matrix protein M2. ACS Synth Biol.

[5] Eggink D (2014). Guiding the immune response against influenza virus hemagglutinin toward the conserved stalk domain by hyperglycosylation of the globular head domain. J Virol.

[6] Morgan SB (2016). Aerosol delivery of a candidate universal influenza vaccine reduces viral load in pigs challenged with pandemic H1N1 virus. J Immunol.

[7] Memoli MJ (2020). Influenza A reinfection in sequential human challenge: implications for protective immunity and “universal” vaccine development. Clin Infect Dis.

[8] Honce R, Schultz-Cherry S (2019). Impact of obesity on influenza A virus pathogenesis, immune response, and evolution. Front Immunol.

[9] Pulendran B (2014). Systems vaccinology: probing humanity’s diverse immune systems with vaccines. Proc Natl Acad Sci U S A.

[10] Gounder AP, Boon ACM (2019). Influenza pathogenesis: the effect of host factors on severity of disease. J Immunol.

[11] Chen J (2020). Role of aging and the immune response to respiratory viral infections: potential implications for COVID-19. J Immunol.

[12] Pop-Vicas A, Gravenstein S (2011). Influenza in the elderly: a mini-review. Gerontology.

[13] Osterholm MT (2012). Efficacy and effectiveness of influenza vaccines: a systematic review and meta-analysis. Lancet Infect Dis.

[14] van der Made CI (2020). Presence of genetic variants among young men with severe COVID-19. JAMA.

[15] Tuite A, Gros P (2006). The impact of genomics on the analysis of host resistance to infectious disease. Microbes Infect.

[16] Trammell RA, Toth LA (2008). Genetic susceptibility and resistance to influenza infection and disease in humans and mice. Expert Rev Mol Diagn.

[17] Querec TD (2009). Systems biology approach predicts immunogenicity of the yellow fever vaccine in humans. Nat Immunol.

[18] Nakaya HI (2011). Systems biology of vaccination for seasonal influenza in humans. Nat Immunol.

[19] Bendall SC (2011). Single-cell mass cytometry of differential immune and drug responses across a human hematopoietic continuum. Science.

[20] Kimball AK (2018). A beginner’s guide to analyzing and visualizing mass cytometry data. J Immunol.

[21] Bendall SC (2012). A deep profiler’s guide to cytometry. Trends Immunol.

[22] Aghaeepour N (2013). Critical assessment of automated flow cytometry data analysis techniques. Nat Methods.

[23] Morales-Nebreda L (2021). Aging imparts cell-autonomous dysfunction to regulatory T cells during recovery from influenza pneumonia. JCI Insight.

[24] Kulkarni U (2019). Excessive neutrophil levels in the lung underlie the age-associated increase in influenza mortality. Mucosal Immunol.

[25] Smith CA (2019). Influenza virus inoculum volume is critical to elucidate age-dependent mortality in mice. Aging Cell.

[26] Toapanta FR, Ross TM (2009). Impaired immune responses in the lungs of aged mice following influenza infection. Respir Res.

[27] Ayala VI (2011). Bordetella pertussis infection exacerbates influenza virus infection through pertussis toxin-mediated suppression of innate immunity. PLoS One.

[28] Srivastava B (2009). Host genetic background strongly influences the response to influenza a virus infections. PLoS One.

[29] Ding M (2008). Gene expression in lung and basal forebrain during influenza infection in mice. Genes Brain Behav.

[30] Boon ACM (2011). H5N1 influenza virus pathogenesis in genetically diverse mice is mediated at the level of viral load. mBio.

[31] Zhao G (2014). Differences in the pathogenicity and inflammatory responses induced by avian influenza A/H7N9 virus infection in BALB/c and C57BL/6 mouse models. PLoS One.

[32] Vapnik V (1997). Support vector method for function approximation, regression estimation, and signal processing. Adv Neural Inf Process Syst.

[33] Abdelaal T (2019). Predicting cell populations in single cell mass cytometry data. Cytometry A.

[34] Amir EAD (2013). viSNE enables visualization of high dimensional single-cell data and reveals phenotypic heterogeneity of leukemia. Nat Biotechnol.

[35] Geissmann F (2010). Development of monocytes, macrophages, and dendritic cells. Science.

[36] Chen X (2018). Host immune response to influenza A virus infection. Front Immunol.

[37] Rojas JM (2017). IL-10: a multifunctional cytokine in viral infections. J Immunol Res.

[38] Boivin WA (2009). Intracellular versus extracellular granzyme B in immunity and disease: challenging the dogma. Lab Invest.

[39] Luzina IG (2012). Regulation of inflammation by interleukin-4: a review of “alternatives”. J Leukoc Biol.

[40] Sun J (2009). Effector T cells control lung inflammation during acute influenza virus infection by producing IL-10. Nat Med.

[41] Bahadoran A (2016). Immune responses to influenza virus and its correlation to age and inherited factors. Front Microbiol.

[42] Manicassamy B (2010). Analysis of in vivo dynamics of influenza virus infection in mice using a GFP reporter virus. Proc Natl Acad Sci U S A.

[43] Steuerman Y (2018). Dissection of influenza infection in vivo by single-cell RNA sequencing. Cell Syst.

[44] Frensing T (2016). Influenza virus intracellular replication dynamics, release kinetics, and particle morphology during propagation in MDCK cells. Appl Microbiol Biotechnol.

[45] Marvin SA (2017). Influenza virus overcomes cellular blocks to productively replicate, impacting macrophage function. J Virol.

[46] Fujimoto I (2000). Virus clearance through apoptosis-dependent phagocytosis of influenza A virus-infected cells by macrophages. J Virol.

[47] Perez-Caballero D (2009). Tetherin inhibits HIV-1 release by directly tethering virions to cells. Cell.

[48] Pinchuk LM, Filipov NM (2008). Differential effects of age on circulating and splenic leukocyte populations in C57BL/6 and BALB/c male mice. Immun Ageing.

[49] Mühl H, Pfeilschifter J (2003). Anti-inflammatory properties of pro-inflammatory interferon-gamma. Int Immunopharmacol.

[50] Kang S (2018). Direct antiviral mechanisms of interferon-gamma. Immune Netw.

[51] Flaishon L (2002). Cutting edge: anti-inflammatory properties of low levels of IFN-gamma. J Immunol.

[52] Kelchtermans H (2008). How interferon-gamma keeps autoimmune diseases in check. Trends Immunol.

[53] Wong CK (2017). Aging impairs alveolar macrophage phagocytosis and increases influenza-induced mortality in mice. J Immunol.

[54] Penke LR (2020). PGE_2_ accounts for bidirectional changes in alveolar macrophage self-renewal with aging and smoking. Life Sci Alliance.

[55] Hufford MM (2015). The effector T cell response to influenza infection. Curr Top Microbiol Immunol.

[56] Zhao J (2011). Age-related increases in PGD(2) expression impair respiratory DC migration, resulting in diminished T cell responses upon respiratory virus infection in mice. J Clin Invest.

[57] Franceschi C (2018). Inflammaging: a new immune-metabolic viewpoint for age-related diseases. Nat Rev Endocrinol.

[58] Lu Y (2013). The interaction of influenza H5N1 viral hemagglutinin with sialic acid receptors leads to the activation of human γδ T cells. Cell Mol Immunol.

[59] Brandes M (2005). Professional antigen-presentation function by human gammadelta T cells. Science.

[60] Qin G (2011). Type 1 responses of human Vγ9Vδ2 T cells to influenza A viruses. J Virol.

[61] Samarasinghe AE (2017). Eosinophils promote antiviral immunity in mice infected with influenza A virus. J Immunol.

[62] Rosenberg HF (2009). Eosinophils and their interactions with respiratory virus pathogens. Immunol Res.

[63] LeMessurier KS (2020). Influenza A virus directly modulates mouse eosinophil responses. J Leukoc Biol.

[64] Goldberg EL (2019). Ketogenic diet activates protective γδ T cell responses against influenza virus infection. Sci Immunol.

[65] Nolz JC (2011). Naive, effector and memory CD8 T-cell trafficking: parallels and distinctions. Immunotherapy.

[66] Parra D (2012). Pivotal advance: peritoneal cavity B-1 B cells have phagocytic and microbicidal capacities and present phagocytosed antigen to CD4^+^ T cells. J Leukoc Biol.

[67] Martínez-Riaño A (2018). Antigen phagocytosis by B cells is required for a potent humoral response. EMBO Rep.

[68] Nakashima M (2012). Pivotal advance: characterization of mouse liver phagocytic B cells in innate immunity. J Leukoc Biol.

[69] Wang X (2007). The interferon-inducible protein viperin inhibits influenza virus release by perturbing lipid rafts. Cell Host Microbe.

[70] Goronzy JJ, Weyand CM (2019). Mechanisms underlying T cell ageing. Nat Rev Immunol.

[71] Howes A (2016). Differential production of type I IFN determines the reciprocal levels of IL-10 and proinflammatory cytokines produced by C57BL/6 and BALB/c macrophages. J Immunol.

[72] Sathyanesan M (2017). Restraint stress differentially regulates inflammation and glutamate receptor gene expression in the hippocampus of C57BL/6 and BALB/c mice. Stress.

[73] Redford PS (2011). The role of IL-10 in immune regulation during M. tuberculosis infection. Mucosal Immunol.

[74] Billi AC (2019). The female-biased factor VGLL3 drives cutaneous and systemic autoimmunity. JCI Insight.

[75] Chevrier S (2018). Compensation of signal spillover in suspension and imaging mass cytometry. Cell Syst.

[76] Bayne K (1996). Revised guide for the care and use of laboratory animals available. American Physiological Society. Physiologist.

