# Supplementary material for: iGATE analysis improves the interpretability of single-cell immune landscape of influenza infection
Source: JCI Insight. 2024 May 30;9(12):e172140. doi: 10.1172/jci.insight.172140 (PMC11383363; doi:10.1172/jci.insight.172140)
Supplement: Supplemental data [file jciinsight-9-172140-s059.pdf]

iGATE analysis improves the interpretability of single-cell immune landscape of influenza infection

Brett D. Hill[1], Andrew J. Zak*[1], Sanjeev Raja[1], Luke F. Bugada[1], Syed M. Rizvi[1], Saiful B. Roslan[1], Hong Nhi Nguyen[1], Judy Chen [2,3], Hui Jiang[4], Akira Ono[5], Daniel R. Goldstein[3], Fei Wen**[1]*

Supplemental Material

Supplemental Methods

List of Abbreviations

Supplemental Table 1

Supplemental Figures 1-13

Supplemental Methods
SVM Classifier

We used linear kernel SVM, as it has been demonstrated that linear decision boundaries are sufficient to achieve excellent class separability in CyTOF data (33). Furthermore, linear models are less susceptible to overfitting that is often encountered with more complex supervised learners such as neural networks. The cleaned dataset was transformed using the inverse hyperbolic sine function and normalized to obtain a per-channel zero mean and unit variance. Multiclass SVM was implemented as a one-versus-one ensemble of binary SVM classifiers. For each learner, one class (*i.e,* cell type) is positive, another is negative, and the rest are ignored. This design exhausts all combinations of class pair assignments for a total of 300 binary learners. To perform inference on a new test sample, all learners are queried, and the final prediction is obtained via majority vote.

In order to identify samples which do not belong to any of the predefined classes, we fitted optimal classification score-to-posterior probability transformation functions for each learner (77). If two classes are perfectly separable, the optimal transformation function is the following step function, which transforms the score s_j corresponding to observation j to a positive class posterior probability

$$P(s_j) = \begin{cases} 0; & s_j < \max\limits_{y_k=-1} s_k \\ \pi; & \max\limits_{y_k=-1} s_k \leq s_j \leq \min\limits_{y_k=+1} s_k \\ 1; & s_j > \min\limits_{y_k=+1} s_k \end{cases}$$

where π is the prior probability that an observation is in the positive class and y_k are binary class labels. If the classes are not separable, the optimal transformation is the sigmoid function

$$P(s_j) = \frac{1}{1 + \exp{(As_j + B)}}$$

where parameters A and B are obtained by Platt's fitting method, a maximum likelihood technique which operates on the same training data used to train the original classifier (77).

With the optimal score transformation function obtained for each learner, we generated a prediction matrix L and a positive class posterior probability matrix P. Both matrices have dimensionality J x K, where J is the number of cells in the dataset and K is the number of binary learners. Final class labels for each cell were obtained by a majority vote of all 300 binary learners (i.e. the numerical mode of each row in L), and final posterior probabilities were obtained by averaging those columns of P corresponding to learners which voted for the winning class label. Cells whose posterior probability fell below a threshold were rejected and placed in the "Other" class. We chose a threshold of 0.995, which corresponds to ~6% of cells being rejected in our dataset. This threshold is consistent with the empirical observation that 5-10% of cells were unable to be assigned to a user-defined cell gate.

List of Abbreviations

AM	Alveolar macrophage
BAL	Bronchoalveolar lavage
BalbY	Young (2 month) BALB/c mice
C57A	Aged (18 month) C57BL/6 mice
C57Y	Young (2 month) C57BL/6 mice
CM	Central memory
DPI	Days post infection
DC	Dendritic cell
DN	Double negative ($CD4^-$ and $CD8^-$)
eM	Exudative macrophage
EM	Effector memory
GzmB	Granzyme B
HA	Hemagglutinin
IFNγ	Interferon gamma
iGATE	In-silico gating training annotating elucidating
iHA	Intracellular-stained HA
hpi	Hours post infection
IL	Interleukin
ILC	Innate lymphoid cell
iM	Interstitial macrophage
Mo	Monocyte
NK	Natural killer cell
NKT	Natural killer T cell
PC	Principal component
PCA	Principal component analysis
Q1	iHA^-sHA^+
Q2	iHA^+sHA^+
Q3	iHA^+sHA^-
Q4	iHA^-sHA^-
sHA	Surface-stained HA
SVM	Support vector machine
Tɣδ	Gamma delta T cell
TNFα	Tumor necrosis factor alpha
viSNE	Visualization of t-distributed stochastic neighbor embedding (t-SNE)

Supplemental Table 1: CyTOF antibody table

	Label	Target	Clone	Company	Cat No
1	112Cd	CD19	6D5	Life Technologies	Q10379
2	141Pr	IFNg	XMG1.2	Biolegend	505812
3	142Nd	TNFalpha	MP6-XT22	Biolegend	506302
4	143Nd	NP	H16-L10-4R5 (HB65)	BioXcell	BE0159
5	144Nd	Siglec-F	E50-2440	BD Biosciences	552125
6	145Nd	CD4	RM4-5	Biolegend	100520
7	146Nd	CD45R (B220)	RA3-6B2	Biolegend	103202
8	147Sm	CD206	C068C2	Biolegend	141701
9	148Nd	CD103	2E7	Biolegend	121402
10	149Sm	CD8	53-6.7	Biolegend	100716
11	150Nd	PDCA-1 (CD317)	129C1	Biolegend	127102
12	151Eu	CD49b	DX5	Biolegend	108902
13	152Sm	Ly6C	HK1.4	Novus	NBP1-28046
14	153Eu	Intracellular HA (iHA)	2F1A7	Sino Biologics	11684-MM03
15	154Sm	CD11c	N418	Biolegend	117302
16	155Gd	I-A/I-E	M5/114.15.2	Biolegend	107602
17	156Gd	CD25	3C7	Biolegend	101902
18	158Gd	IgM	RMM-1	Biolegend	406527
19	159Tb	Ly6G	1A8	Biolegend	127637
20	160Gd	IL-4	11B11	Biolegend	504102
21	161Dy	Surface HA (sHA)	2F1A7	Sino Biologics	11684-MM03
22	162Dy	TCR γ/δ	GL3	Biolegend	118101
23	163Dy	CD64	X54-5/7.1	Biolegend	139302
24	164Dy	IL-10	JES5-16E3	Biolegend	505002
25	165Ho	CD49d	9C10 (MFR4.B)	Biolegend	103708
26	166Er	CXCR5	614641	Novus Biologicals	MAB6198
27	167Er	CD127	A7R34	Biolegend	135002
28	168Er	CD24	M1/69	Biolegend	101829
29	169Tm	Ki67	16A8	Biolegend	652402
30	170Er	CD62L	MEL-14	Biolegend	104402
31	171Yb	CD44	IM7	Biolegend	103014
32	172Yb	CD11b	M1/70	Fluidigm	3172012B
33	173Yb	CD69	H1.2F3	Biolegend	104502
34	174Yb	IgD	11-26c.2a	Biolegend	405702
35	175Lu	KLRG1	2F1	Novus Biologicals	MAB69441-100
36	176Yb	Granzyme B	GB11	Abcam	ab10912
37	191/193Ir	DNA		Fluidigm	201192B
38	195Pt	Live/Dead		Fluidigm	201064
39	209Bi	CD3	145-2C11	Biolegend	100314
40	89Y	mCD45	30-F11	Fluidigm	3089005B

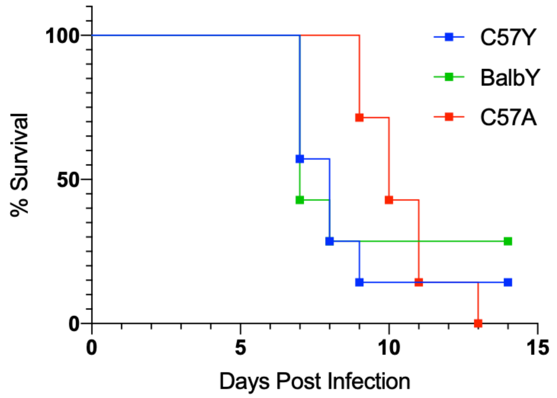

Supplemental Figure 1
Survival following influenza viral infection with 2.0×10^4 plaque forming units (PFU) of H1N1 A/PR/8/34 (n = 7 per group, 1 experiment).

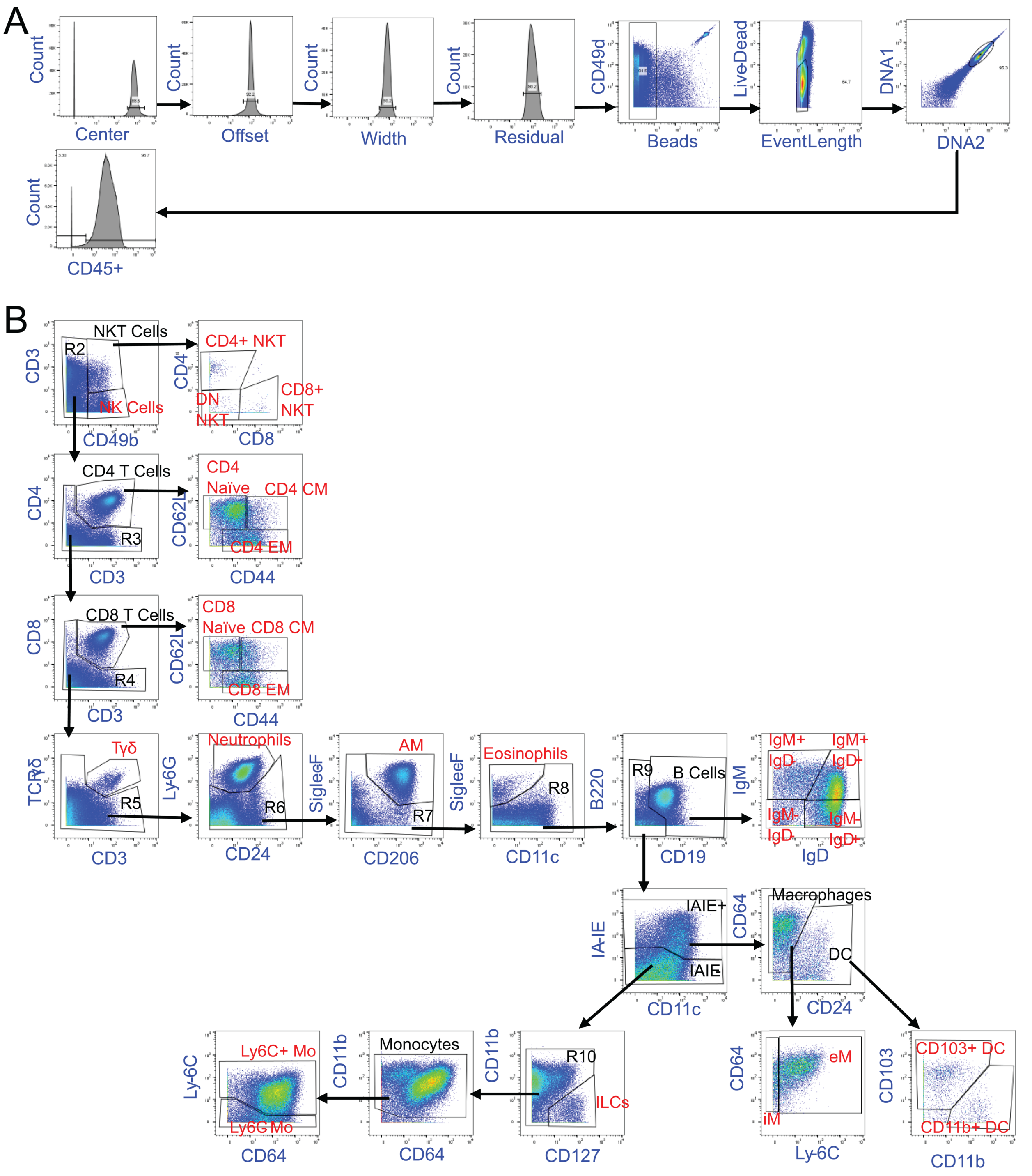

Supplemental Figure 2

Gating strategy to identify 25 canonical immune cell types. (**A**) All events were manually gated to obtain CD45^{+} live singlets using FlowJo (FlowJo, LLC, Ashland, OR). The resulting 29 million CD45^{+} live singlets were randomly and equally sampled from each treatment group to generate a small dataset of 350,000 cells. (**B**) These 350,000 cells were used to define the 25 canonical immune cell types by manual gating.

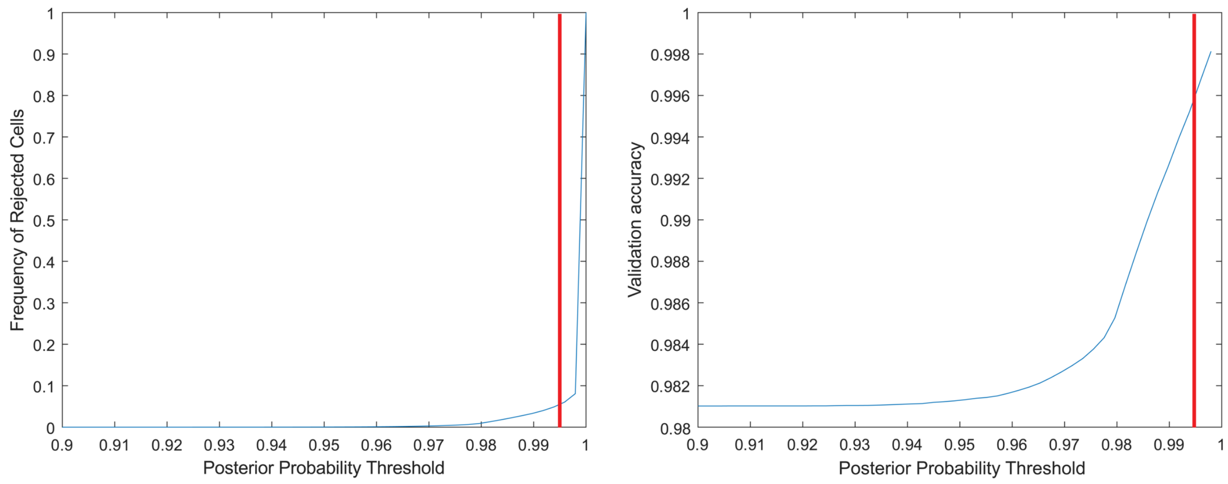

Supplemental Figure 3

Effect of posterior probability threshold on proportion of rejected cells (left) and validation accuracy (right). Red line indicates the chosen posterior probability threshold of 0.995.

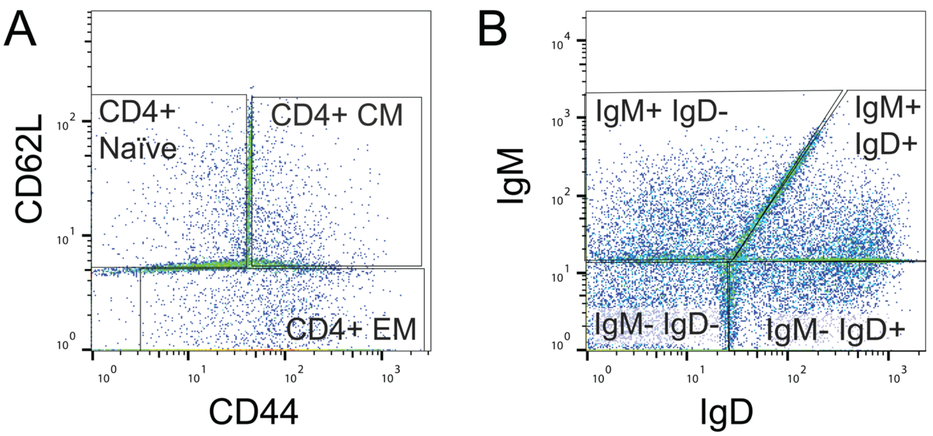

Supplemental Figure 4

Rejected classifier cells predominantly exist on gate boundaries as shown for rejected CD4$^+$ T cell subsets (**A**) and B cell subsets (**B**).

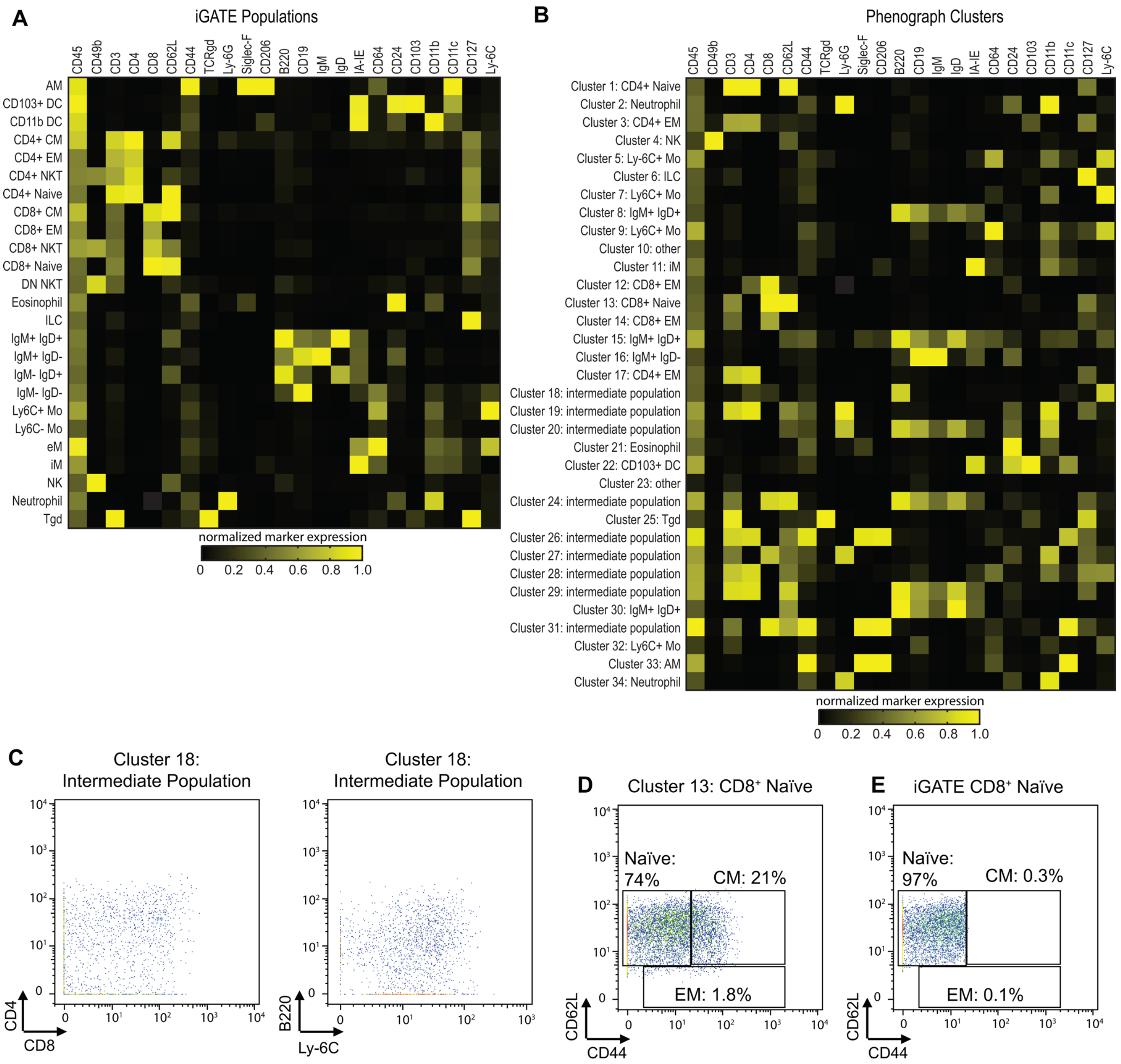

Supplemental Figure 5

Heatmaps showing marker expression of (**A**) iGATE and (**B**) Phenograph populations. "Intermediate population" indicates clusters that could not be defined as canonical populations. Despite generating 34 Phenograph clusters, 10 of the 25 canonical cell populations defined in **Table 1** were missed by Phenograph: $CD4^+$ NKT, $CD8^+$ NKT, DN NKT, IgM^-IgD^+ B cells, IgM^-IgD^- B cells, eM, $CD4^+$ CM T cells, $CD8^+$ CM T cells, $CD11b^+$ DCs, and $Ly6C^-$ Mo. (**C**) Biplots of Phenograph cluster 18, an intermediate population, showing CD4, CD8, B220, and Ly-6C confirm that cluster 18 is a heterogenous mixture of cell populations. iGATE populations are more homogenous with fewer incorrectly classified cells compared to corresponding populations identified by Phenograph. Biplots of CD62L and CD44 expression demonstrate that (**D**) only 74% of the Phenograph $CD8^+$ Naïve T cells (Cluster 13) are correctly labeled, while (**E**) 97% of the iGATE $CD8^+$ Naïve T cells are correctly labeled according to the gating scheme in **Supplemental Figure 2B**.

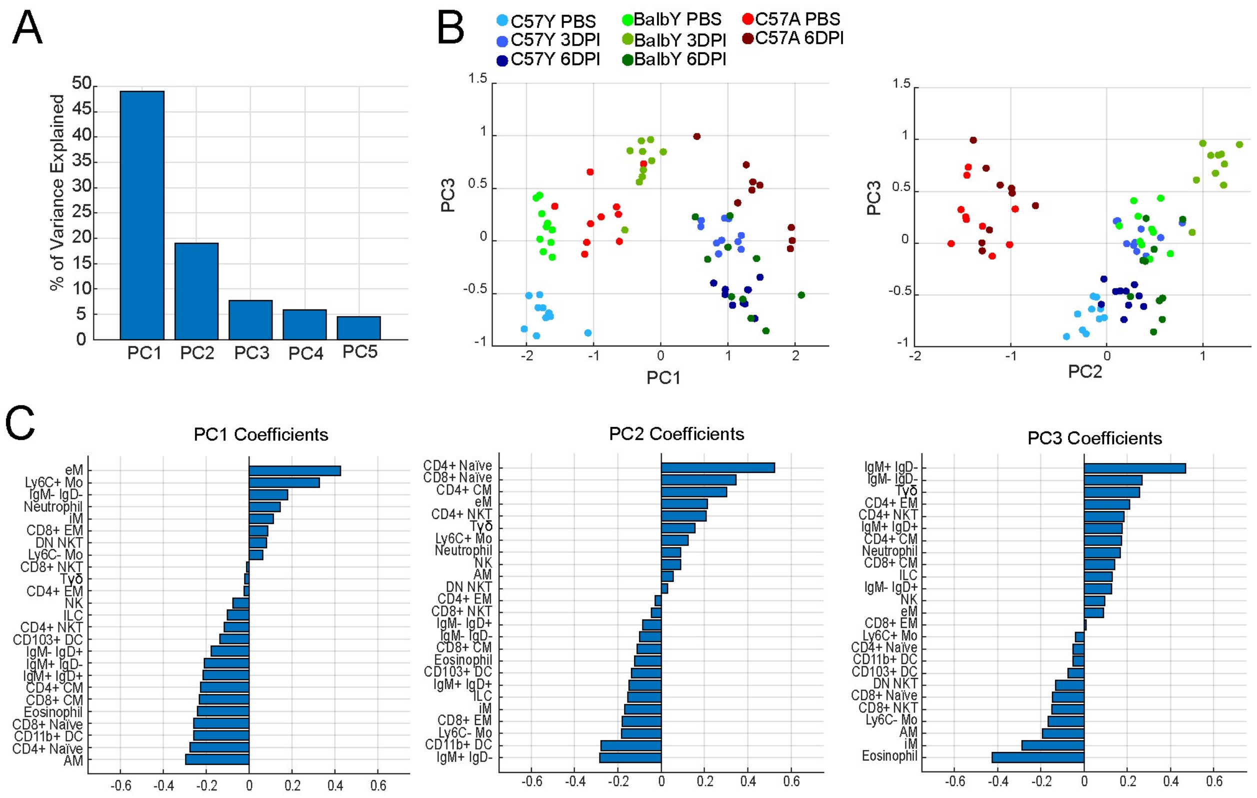

Supplemental Figure 6

(**A**) Percentage of variance explained by principal components (PCs). (**B**) 2-dimensional plots of PC3 vs. PC1 and PC3 vs. PC2. (**C**) PC coefficients.

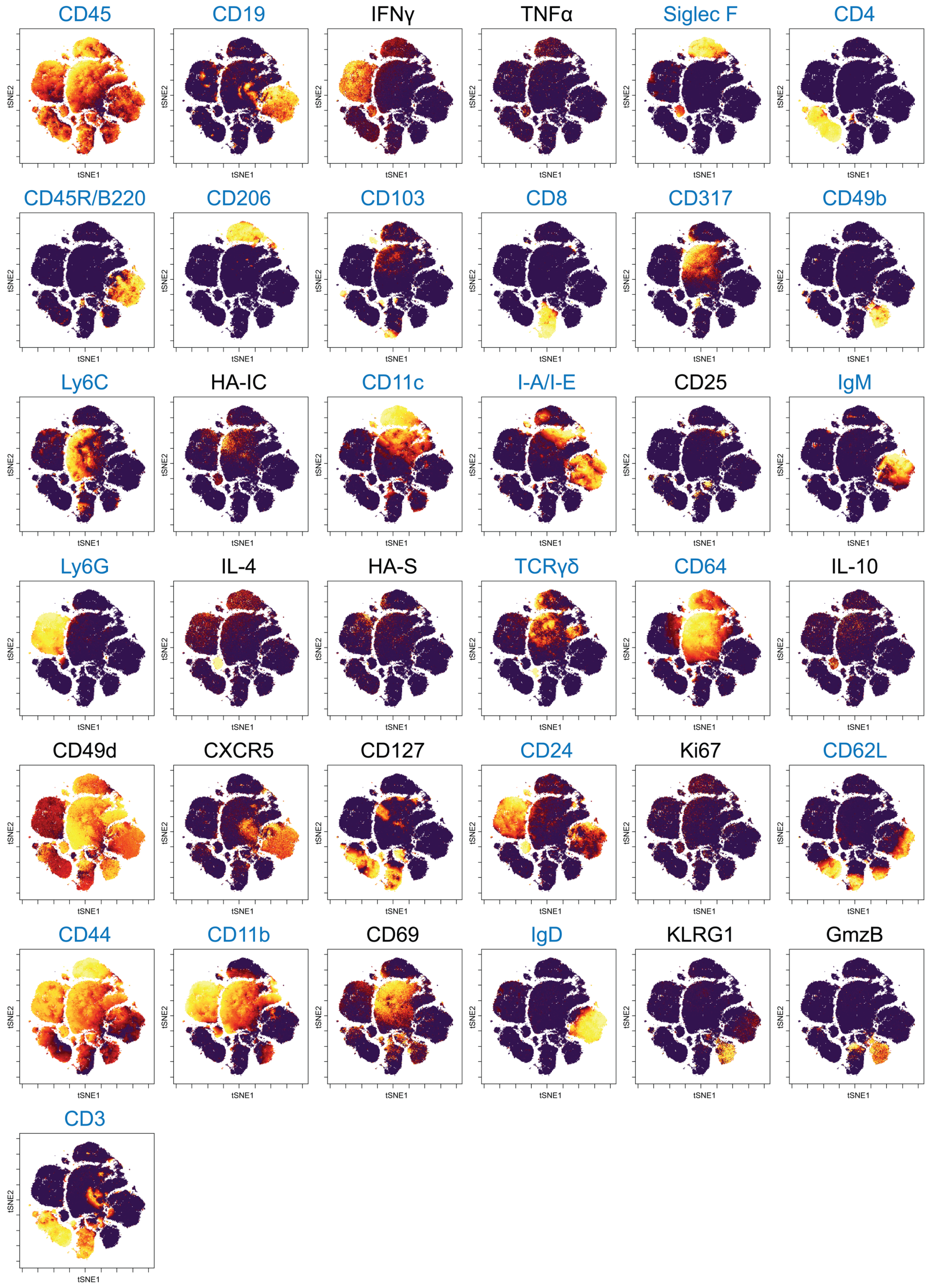

Supplemental Figure 7

Heatmaps of marker expression overlaid on the viSNE map. ViSNE analysis was performed on 250,000 cells sampled from all groups based on markers colored in blue.

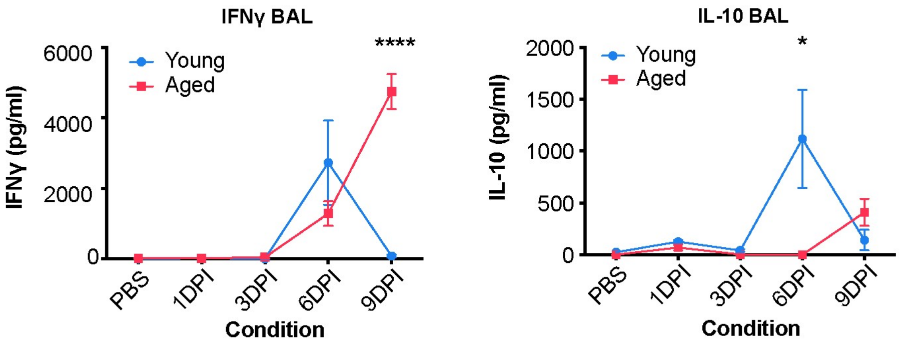

Supplemental Figure 8

Cytokine levels in BAL at different days post infection (DPI) measured by ELISA. A spike in cytokine TNFα at 6DPI in a similar experimental system has been published elsewhere (24). BAL was collected by lavaging the lungs with 1 mL of ice-cold PBS. BAL was aliquoted and then stored at -80°C until analysis. ELISA was performed following manufacturer's directions. ELISA kits used: IFNγ (Invitrogen 88-7314-22) and IL-10 (Invitrogen 88-7015-22). Each data point represents the mean ± SEM of 2-5 mice. Statistical comparisons were computed with Student's *t* test with FDR of 10%. *$q < 0.10$, ****$q < 0.0001$.

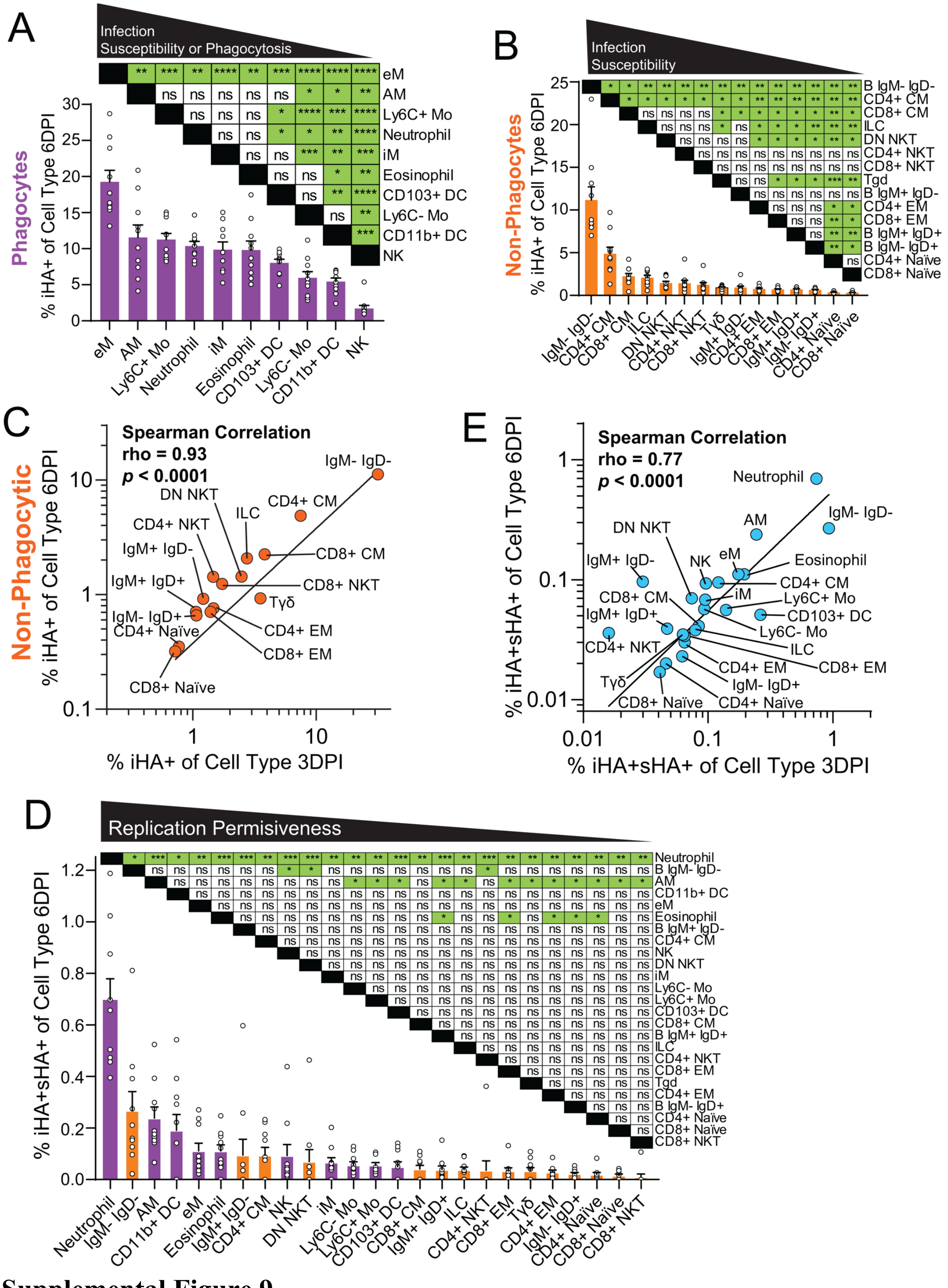

Supplemental Figure 9

%iHA$^+$ cells by cell type for (**A**) phagocytes and (**B**) non-phagocytes in C57Y 6DPI. (**C**) Comparison of mean %iHA$^+$ of non-phagocytic cells in C57Y between 3DPI and 6DPI. (**D**) %iHA$^+$sHA$^+$ cells by cell type for phagocytes (purple) and non-phagocytes (orange) in C57Y 6DPI. (**E**) Comparison of mean %iHA$^+$sHA$^+$ of all immune cell types between 3DPI and 6DPI in C57Y. Statistical comparisons in **C** and **E** were computed by Spearman correlation, and a linear regression line is shown. Data represent mean ± SEM, n = 10; statistical comparisons in A, B, and D were computed by paired one-way ANOVA with post hoc Tukey's pairwise comparisons with *q < 0.10, **q < 0.01, ***q < 0.001, ****q < 0.0001.

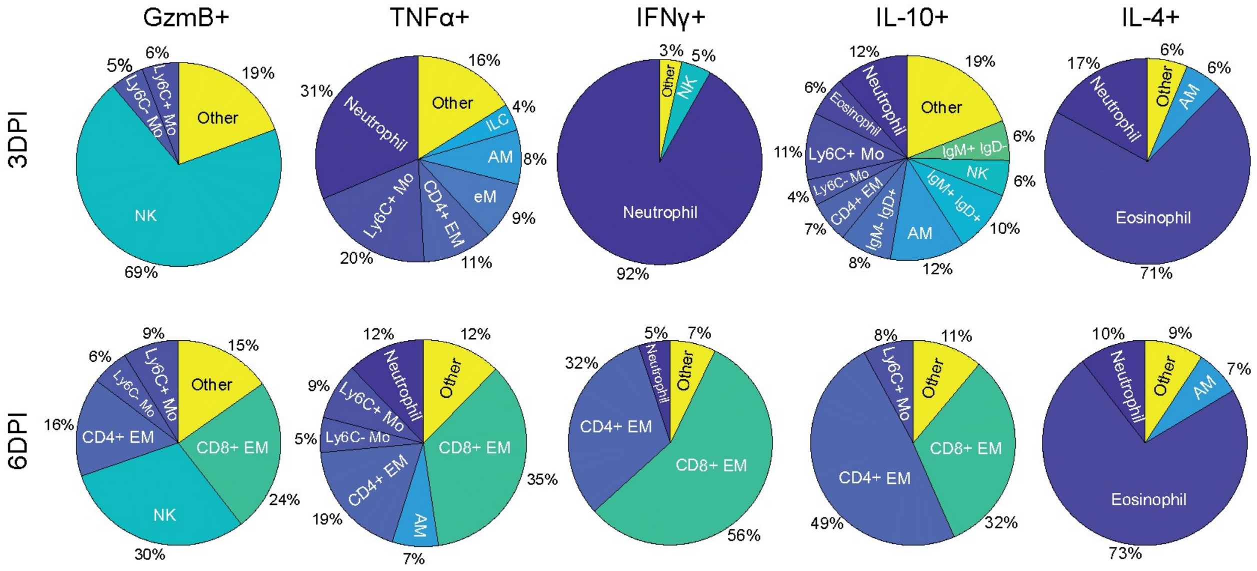

Supplemental Figure 10

Contribution of indicated immune cell types to total cytokine positive cells in BalbY at 3DPI and 6DPI. Contributions < 4% are grouped into "other". Data represent n = 10 per group.

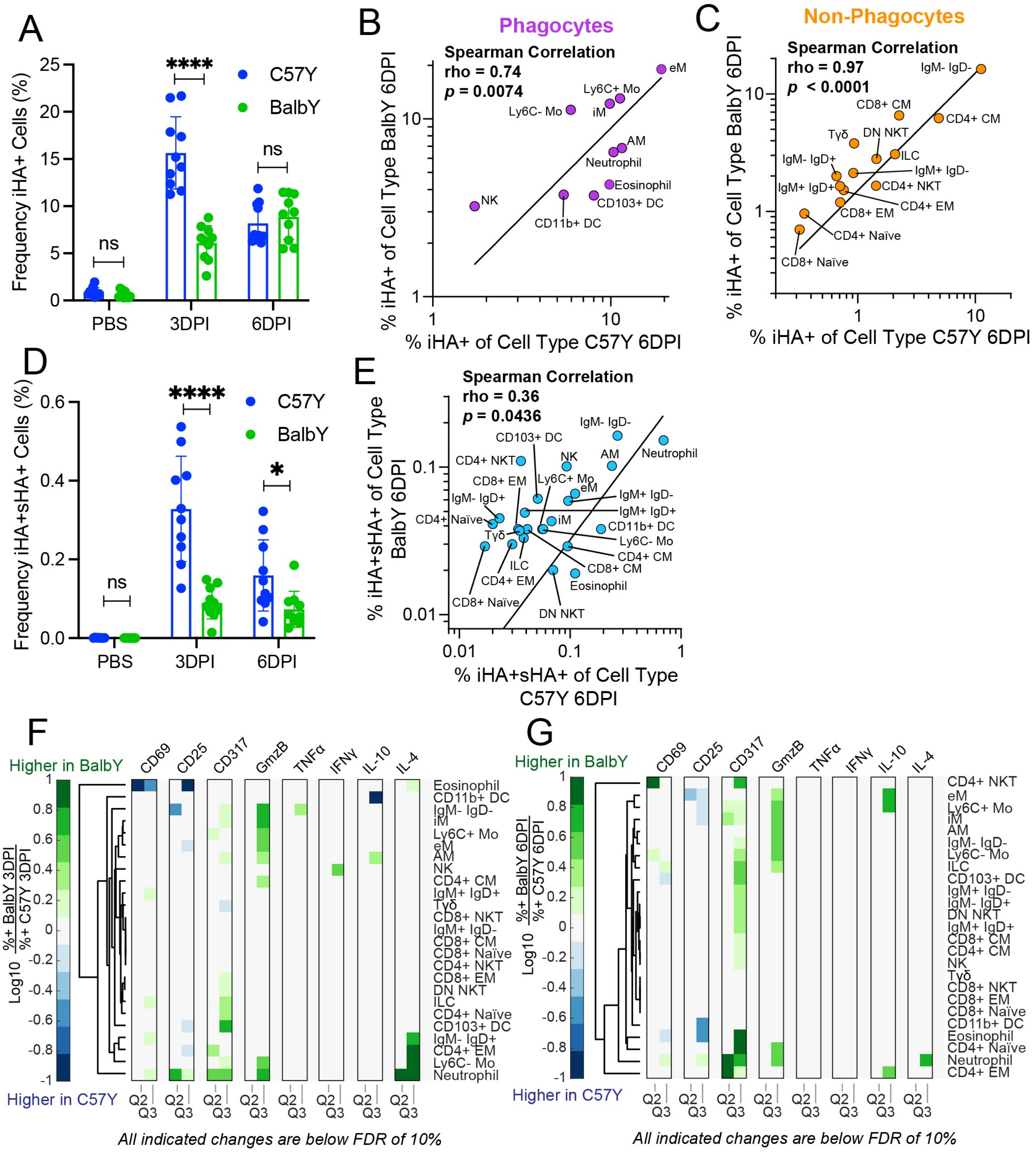

Supplemental Figure 11

(**A**) Comparison of the frequency of iHA$^+$ cells between C57Y and BalbY (n = 10). Comparison of %iHA$^+$ for each cell type for (**B**) phagocytes and (**C**) non-phagocytes at 6DPI between C57Y and BalbY. (**D**) Comparison of the frequency of iHA$^+$sHA$^+$ cells between C57Y and BalbY. (**E**) Comparison of the %iHA$^+$sHA$^+$ cells for all immune cell types between C57Y and BalbY at 6DPI. (**F, G**) Log fold change in marker expression between C57Y and BalbY mice for indicated immune cell types in Q2 (iHA$^+$sHA$^+$) and Q3 (iHA$^+$sHA$^-$) at 3DPI (**F**) and 6DPI (**G**). All data represent mean ± SEM, n = 10; statistical comparisons in **A** and **D** were computed by Student's t test with FDR of 10%, *q < 0.10, ****q < 0.0001; statistical comparisons in **B**, **C**, and **E** were computed by Spearman correlation, and a linear regression line is also shown.

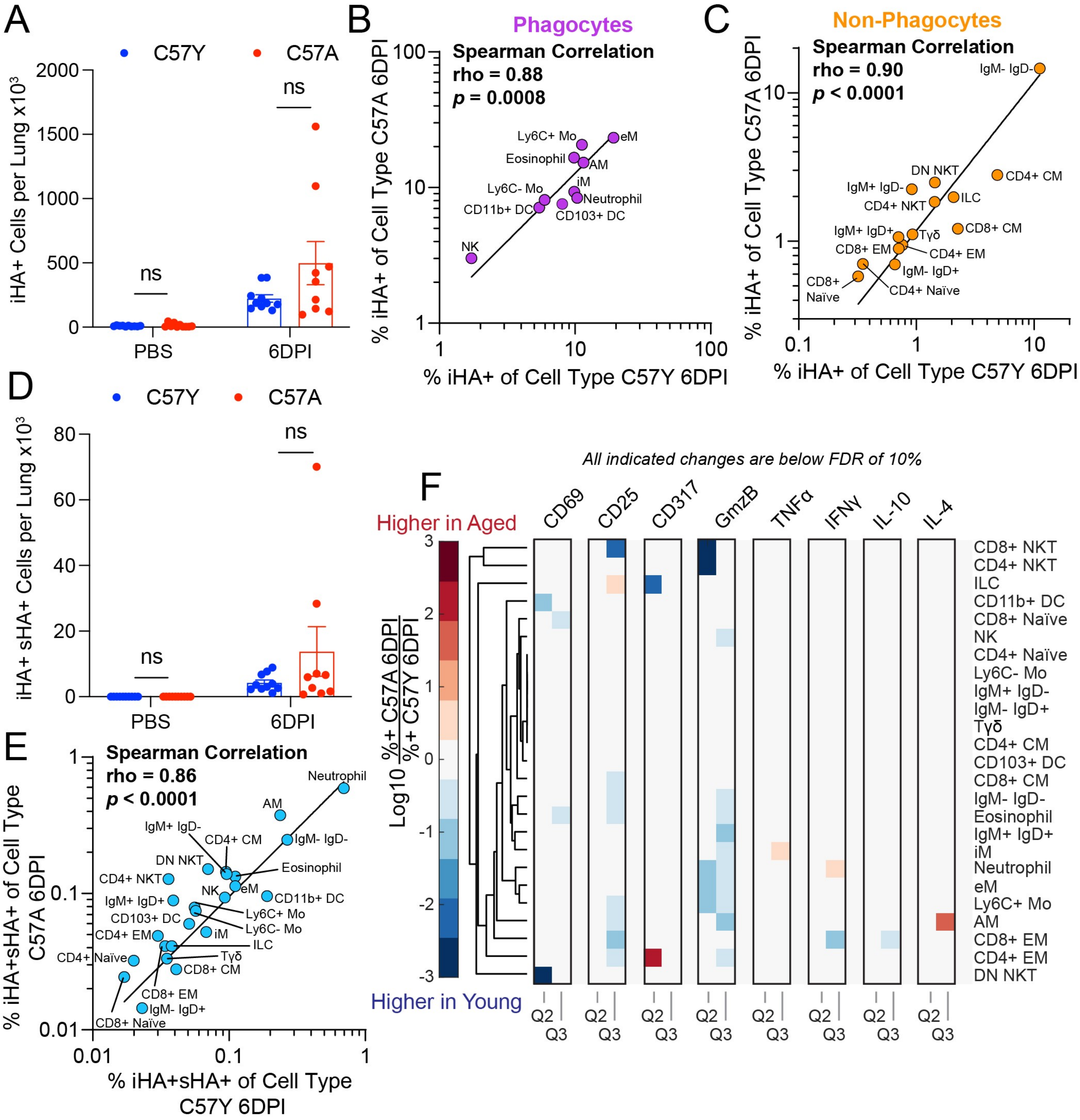

Supplemental Figure 12

(**A**) Comparison of iHA$^+$ cell counts between C57Y and C57A. Comparison of %iHA$^+$ of each cell type at 6DPI for (**B**) phagocytes and (**C**) non-phagocytes between C57Y and C57A. Indicated significance is a result of Spearman correlation and a linear regression line is shown. (**D**) Comparison of iHA$^+$sHA$^+$ cell counts between C57Y and C57A. (**E**) Comparison of %iHA$^+$sHA$^+$ of all immune cell types between C57Y and C57A. Indicated significance is a result of Spearman correlation and a linear regression line is shown. (**F**) Log fold change of the fraction of functional marker expression between C57Y and C57A for indicated immune cell types in Q2 (iHA$^+$sHA$^+$) and Q3 (iHA$^+$sHA$^-$). Data in **A** and **D** represent mean ± SEM. C57Y PBS n = 10, C57A PBS n = 10, C57Y 6DPI n = 10, C57A 6DPI n = 9. Statistical comparisons were computed by two-sided Student's t test with FDR = 10%.

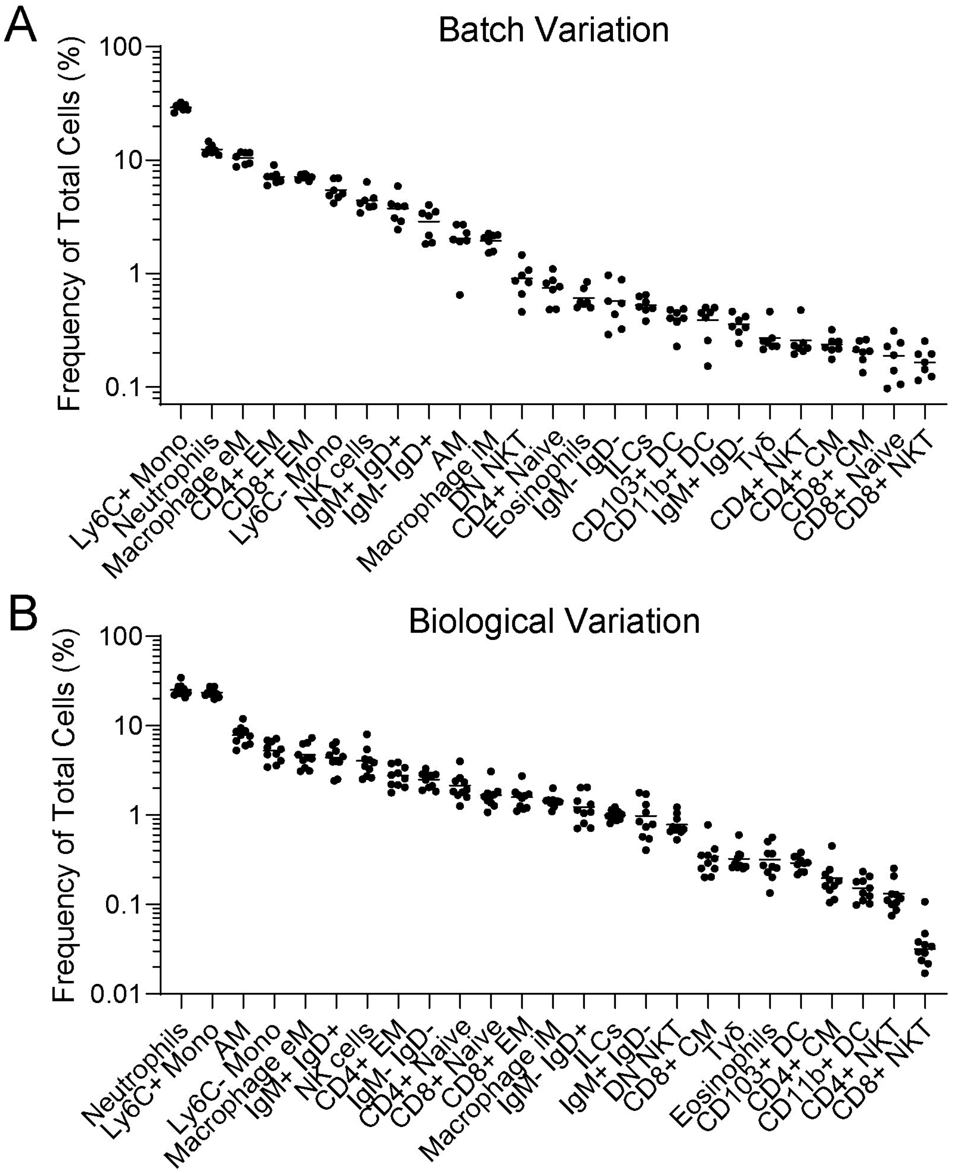

Supplemental Figure 13: Frequency of each immune cell types in control samples

(**A**) CyTOF sample acquisition was divided into seven batches. Along with each batch, an aliquot of a frozen control sample (representing infected C57Y mouse lung homogenate) was included. (**B**) Immune cell frequencies were plotted for each C57Y 3DPI mouse (n = 10) to evaluate biological variation. Comparison of the seven control samples in (**A**) and the 10 C57Y 3DPI samples in (**B**) demonstrates tight distributions for all 25 immune cell types, indicating minimal batch-to-batch and biological variation.
